# In vivo protein kinase activity of SnRK1 fluctuates in Arabidopsis rosettes during light-dark cycles

**DOI:** 10.1093/plphys/kiad066

**Published:** 2023-02-02

**Authors:** Omri Avidan, Thiago A Moraes, Virginie Mengin, Regina Feil, Filip Rolland, Mark Stitt, John E Lunn

**Affiliations:** Metabolic Networks, Max Planck Institute of Molecular Plant Physiology, Am Mühlenberg 1, 14476 Potsdam-Golm, Germany; Metabolic Networks, Max Planck Institute of Molecular Plant Physiology, Am Mühlenberg 1, 14476 Potsdam-Golm, Germany; Metabolic Networks, Max Planck Institute of Molecular Plant Physiology, Am Mühlenberg 1, 14476 Potsdam-Golm, Germany; Metabolic Networks, Max Planck Institute of Molecular Plant Physiology, Am Mühlenberg 1, 14476 Potsdam-Golm, Germany; Laboratory of Molecular Plant Biology, KU Leuven, B-3001 Leuven, Belgium; KU Leuven Plant Institute (LPI), B-3001 Leuven, Belgium; Metabolic Networks, Max Planck Institute of Molecular Plant Physiology, Am Mühlenberg 1, 14476 Potsdam-Golm, Germany; Metabolic Networks, Max Planck Institute of Molecular Plant Physiology, Am Mühlenberg 1, 14476 Potsdam-Golm, Germany

## Abstract

Sucrose-nonfermenting 1 (SNF1)–related kinase 1 (SnRK1) is a central hub in carbon and energy signaling in plants, and is orthologous with SNF1 in yeast and the AMP-activated protein kinase (AMPK) in animals. Previous studies of SnRK1 relied on in vitro activity assays or monitoring of putative marker gene expression. Neither approach gives unambiguous information about in vivo SnRK1 activity. We have monitored in vivo SnRK1 activity using Arabidopsis (*Arabidopsis thaliana*) reporter lines that express a chimeric polypeptide with an SNF1/SnRK1/AMPK-specific phosphorylation site. We investigated responses during an equinoctial diel cycle and after perturbing this cycle. As expected, in vivo SnRK1 activity rose toward the end of the night and rose even further when the night was extended. Unexpectedly, although sugars rose after dawn, SnRK1 activity did not decline until about 12 h into the light period. The sucrose signal metabolite, trehalose 6-phosphate (Tre6P), has been shown to inhibit SnRK1 in vitro. We introduced the SnRK1 reporter into lines that harbored an inducible *trehalose-6-phosphate synthase* construct. Elevated Tre6P decreased in vivo SnRK1 activity in the light period, but not at the end of the night. Reporter polypeptide phosphorylation was sometimes negatively correlated with Tre6P, but a stronger and more widespread negative correlation was observed with glucose-6-phosphate. We propose that SnRK1 operates within a network that controls carbon utilization and maintains diel sugar homeostasis, that SnRK1 activity is regulated in a context-dependent manner by Tre6P, probably interacting with further inputs including hexose phosphates and the circadian clock, and that SnRK1 signaling is modulated by factors that act downstream of SnRK1.

## Introduction

Plant sucrose-nonfermenting 1–related protein kinase 1 (SnRK1) belongs to a eukaryotic family of Ca^2+^-independent Ser/Thr protein kinases. SnRK1 is implicated in maintaining cellular homeostasis and energy balance alongside linking carbon (C) availability and growth, and has been extensively studied in recent years (Baena-González et al. 2007; Polge and Thomas 2007; Jossier et al. 2009; Hulsmans et al. 2016; Paul et al. 2018, 2020; Crepin and Rolland 2019; Baena-González and Lunn 2020). These functions are broadly similar to those observed for its yeast and animal orthologues, sucrose-nonfermenting 1 (SNF1) and AMP-activated protein kinase (AMPK) but our understanding of the functions and regulation of SnRK1 in plants is much less complete.

SnRK1 is a heterotrimer consisting of a catalytic (α) subunit and two regulatory (β and βγ) subunits that modify complex stability, activity, interactions and cellular localization (Polge et al. 2008; Nietzsche et al. 2016; Wang et al. 2020). It is located in the nucleus and cytosol (Nietzsche et al. 2014; Blanco et al. 2019; Ramon et al. 2019) and has also been reported to be present in plastids (Ávila-Castañeda et al. 2014; Ruiz-Gayosso et al. 2018). According to current knowledge, the mode of regulation differs from that of SNF1 and AMPK. The regulatory properties of these kinases have been studied mainly by in vitro assays, which monitor phosphorylation of peptides containing an evolutionarily conserved SNF1/SnRK1/AMPK-specific recognition sequence: [MLVFI].X.[RKH].X.X.S.X.X.X.[LFIMV] or the less favored [MLVFI].[RKH].X.X.X.S.X.X.X.[LFIMV] (Dale et al. 1995). Such studies have demonstrated that animal AMPKs, and to a certain degree also yeast SNF1, are directly regulated by AMP and/or ADP (Hardie and Carling 1997; Hardie et al. 1998; Mayer et al. 2011). In mammals, these adenylates inhibit T-loop dephosphorylation, thereby activating the catalytic (α) subunit in low energy conditions and/or in response to low C availability (Crozet et al. 2014; García-Salcedo et al. 2014). In contrast, plant SnRK1 is not activated by adenylates in in vitro assays. Instead, it is inhibited in vitro by sugar phosphates, such as trehalose 6-P (Tre6P), glucose-6-phosphate (Glc6P) and glucose 1-phosphate (Glc1P) (Toroser et al. 2000; Lu et al. 2007; Zhang et al. 2009; Nunes et al. 2013b; Emanuelle et al. 2015; Broeckx et al. 2016; Zhai et al. 2017, 2018; Ruiz-Gayosso et al. 2018). Inhibition by sugar phosphates could potentially link SnRK1 activity to C availability rather than energy status (Hara et al. 2013; Nunes et al. 2013b; Herzig and Shaw 2018). These in vitro studies also indicated that inhibition by Tre6P requires an unknown proteinaceous factor that is present in growing tissues but not in mature source leaves (Toroser et al. 2000; Zhang et al. 2009; Emanuelle et al. 2015). Subsequent studies have shown that Tre6P can bind directly to the SnRK1 catalytic α subunit, thereby interfering with binding of the SnRK1-activating kinases GRIK1/2, leading to inhibition of SnRK1 activity (Glab et al. 2017; Zhai et al. 2018; Hwang et al. 2019). However, GRIK1/2 might not be the only mechanism by which Tre6P inhibits SnRK1 activity. As GRIK1/2 are widely expressed in source leaves, Zhai et al. (2018) concluded that they were unlikely to be the unknown proteinaceous factor from young tissues that was referred to by Zhang et al. (2009).

Glc6P and Glc1P are intermediates in glycolysis, gluconeogenesis, and in the synthesis and degradation of major carbohydrate reserves like starch and sucrose. Tre6P is an intermediate in the pathway of trehalose biosynthesis. It is synthesized from UDP-Glc and Glc6P by trehalose-6-phosphate synthase (TPS) and is dephosphorylated to trehalose by trehalose-6-phosphate phosphatase (TPP) (Cabib and Leloir 1958). Trehalose is a major C storage metabolite, osmolyte, and stress protectant in fungi, but has been largely replaced by sucrose in vascular plants, allowing neo-functionalization of the trehalose biosynthesis pathway (Goddijn and van Dun 1999; Lunn et al. 2014; Lunn 2016). In addition to catalytically active Class I TPS proteins, plants possess a large family of catalytically inactive Class II TPS proteins (Leyman et al. 2001; Harthill et al. 2006; Lunn 2007; Vandesteene et al. 2010; Lunn et al. 2014; Delorge et al. 2015) as well as a large family of TPP proteins (Leyman et al. 2001; Vandesteene et al. 2012; Kretzschmar et al. 2015). Tre6P is considered to serve as a signal of sucrose availability (Lunn et al. 2006), acting to regulate C allocation between sucrose and other photosynthesis products in source leaves, and to link sucrose supply and growth in sink organs (Schluepmann et al. 2003; Martins et al. 2013; Wahl et al. 2013; Figueroa et al. 2016; Oszvald et al. 2018; Paul et al. 2018, 2020; Fichtner and Lunn 2021).

The possible contribution of Glc6P and Glc1P to regulation of SnRK1 activity *in planta* has not been addressed in detail. Interactions between Tre6P and SnRK1 have been more extensively studied, mainly by investigating changes in SnRK1 marker transcript abundance (Zhang et al. 2009, 2015; Henry et al. 2015; Bledsoe et al. 2017) and post-translational regulation of target enzymes (Sheen and Baena-González 2008; Figueroa et al. 2016). The former builds on the work of Baena-González et al. (2007) who, using a protoplast overexpression system, identified over 1,000 genes as downstream targets of SnRK1, such as *DARK INDUCIBLE 6* (*DIN6*), *DIN1*, *BRANCHED CHAIN AMINO ACID TRANSFERASE 2* (*BCAT2*), and *EXPANSIN 10* (*EXP10*), whose expression is regulated by SnRK1-mediated phosphorylation of upstream transcription factors, such as BASIC LEUCINE-ZIPPER 11 (bZIP11; Ma et al. 2011) and bZIP63 (Mair et al. 2015). Their expression has been widely used as a sensitive readout of SnRK1 activity, either via promoter activation assays (Rodrigues et al. 2013; Ramon et al. 2019) or by analyses of transcript abundance. Abundance of SnRK1-induced transcripts is typically low in a diel cycle but rises strongly when plants are starved by transfer to continuous darkness (Baena-González et al. 2007; Sheen and Baena-González 2008; Usadel et al. 2008; Flis et al. 2016) as expected if SnRK1 is activated in C starvation conditions (Mair et al. 2015; Pedrotti et al. 2018; Ramon et al. 2019). Their abundance often changes reciprocally to Tre6P levels in starvation treatments and other perturbations in several species and tissues (Martínez-Barajas et al. 2011; Wingler et al. 2012; Nunes et al. 2013a; Henry et al. 2015; Nuccio et al. 2015; Baena-González and Lunn 2020; Peixoto et al. 2021) providing correlative evidence for the idea that Tre6P inhibits SnRK1 in vivo. Application of permeable Tre6P analogues to Arabidopsis (*Arabidopsis thaliana*) led to changes in transcript abundance for a subset of these SnRK1 downstream target genes that were consistent with Tre6P acting to inhibit SnRK1 (Griffiths et al. 2016). Further support for partial convergence of the SnRK1 and Tre6P signaling pathways was provided by the identification of several Class II TPSs as transcriptional and post-translational SnRK1 targets (Glinski and Weckwerth 2005; Harthill et al. 2006; Cho et al. 2016; Nukarinen et al. 2016). However, the functional significance of this observation remains unclear because the precise role of the TPS Class II proteins has not yet been defined (Schluepmann and Paul 2009; Ponnu et al. 2011; Yang et al. 2012; Lunn et al. 2014; Delorge et al. 2015; Figueroa and Lunn 2016). It was recently shown that TPS Class II proteins bind to SnRK1 in vivo, and that when TPS Class II proteins are transiently co-expressed with the SnRK1α1 catalytic subunit, by infiltration of *Nicotiana benthamiana* leaves, they attenuate downstream transcriptional responses to SnRK1 (Van Leene et al. 2022). Furthermore, mutant lines with increased or reduced expression of SnRK1 exhibit an altered relationship between sucrose and Tre6P in diel cycles, as well as modified anaplerotic flux of carbon into the tricarboxylic acid cycle that is reminiscent of responses to Tre6P (Peixoto et al. 2021), and mutations in the *SnRK1α1* and *SnRK1βγ* genes were shown to suppress developmental defects in the Arabidopsis *tps1* mutant (Zacharaki et al. 2022).

Taken together, these results point to SnRK1 and Tre6P signaling being closely intertwined. They also indicate that SnRK1 signaling is involved not only in starvation responses, but also in the coordination of metabolism in benign conditions where plants are not C starved or facing a large C excess (see also Baena-González and Lunn 2020). However, many open questions remain; for example, negative relationships between Tre6P levels and the transcript abundance of SnRK1-induced genes are seen in mature tissues even though Tre6P does not inhibit SnRK1 activity in in vitro assays with material from mature tissues (see above and Baena-González and Lunn 2020). Moreover, there is an inconsistent relationship between Tre6P levels and transcript abundance of SnRK1 target genes in in vitro cultured excised maize (*Zea mays*) kernels (Bledsoe et al. 2017, see also Baena-González and Lunn 2020). It remains possible that Tre6P also acts on signaling downstream of SnRK1, as is the case in yeast (Deroover et al. 2016), resulting in changes in downstream transcript abundance that are not directly due to inhibition of SnRK1 itself. Interpretation is further complicated by potential interactions between SnRK1 and TARGET OF RAPAMYCIN (TOR) signaling (Lastdrager et al. 2014; Margalha and Confraria 2019; Ryabova et al. 2019).

The two main approaches employed in plants to study SnRK1 regulation have shortcomings which have not been addressed to date. The in vitro assay is direct and highly specific, but the use of crude extracts or purified proteins may bring about other complications due to loss of the in vivo context, such as artificial interactions or potential loss of regulatory elements that otherwise would have an effect on activity (Baena-González and Lunn 2020). Monitoring changes in expression of SnRK1 marker genes is sensitive, but gene expression is an indirect readout that may be prone to secondary effects that are unrelated to SnRK1 activity. Indeed, there is often poor agreement between SnRK1 activity in the in vitro phosphorylation assay and changes in SnRK1 target gene expression (Debast et al. 2011; Martínez-Barajas et al. 2011; Baena-González and Lunn 2020).

Working in yeast, Deroover et al. (2016) developed a specific in vivo SNF1 reporter that harbors an SNF1/SnRK1/AMPK-specific recognition sequence-containing peptide, based on rat ACETYL COA CARBOXYLASE 1 (ACC1), an established AMPK target (Dale et al. 1995). The in vivo phosphorylation status of this peptide can be monitored by extraction and immunoblot analysis with a validated commercial phospho-specific antibody, providing a direct measure of in vivo SNF1 activity. Subsequently, modified ACC1 reporters were used in Arabidopsis to study the regulation of SnRK1 activity by nitrogen status (Sanagi et al. 2021) and in other responses (Muralidhara et al. 2021; Henninger et al. 2022; Belda-Palazón et al. 2022).

We have used Arabidopsis ACC1 reporter lines to investigate in vivo SnRK1 activity during a diel cycle in benign conditions when the C supply is only slightly restricting growth, and after changing the light regime to modify the C supply. We have also introduced the reporter construct into Arabidopsis lines in which Tre6P levels can be increased in an inducible manner (Martins et al. 2013; Figueroa et al. 2016). We show that SnRK1 activity does not change reciprocally to overall C availability, with a peak at dawn and a minimum at dusk. Instead, SnRK1 activity appears to be maintained in the light period, declines after darkening and rises toward the end of the night. Furthermore, SnRK1 activity in vivo is not strongly or consistently inhibited by an induced increase in Tre6P. Multiple regression analyses of metabolite levels indicate that the link between in vivo SnRK1 activity and the level of Tre6P is context-dependent and point to SnRK1 activity being regulated in vivo by additional mechanisms, including inhibition by Glc6P.

## Results

### Reporter constructs to quantify in vivo SnRK1 activity

To obtain a direct in vivo readout of SnRK1 activity in Arabidopsis we employed transgenic SnRK1-reporter lines expressing a peptide derived from the Ser79 phosphorylation site of ACC1 fused to green fluorescent protein (GFP), which was used to normalize for the level of expression of the reporter polypeptide (see Supplemental Fig. S1 for full description) and to monitor its localization (see below). The level of phosphorylation of the ACC1 peptide (pACC) was assessed using a specific anti-phospho-site antibody, and an anti-GFP antibody was used to normalize for expression level and gel loading. To increase statistical and biological robustness, we used three to four biological replicates per time point. An additional control sample (extended night) was loaded in all gels, and used to correct for between-gel variation. We took the normalized pACC/GFP signal ratio as a readout of in vivo SnRK1 activity at a given time point (for details see Materials and Methods and Supplemental Fig. S1).

Two transgenic lines were used: (i) expressing a nuclear reporter (NUC), that contains the SV40 nuclear location signal (NLS) and (ii) expressing a general reporter (GEN), in which GFP is observed in both the nucleus and the cytoplasm (Supplemental Fig. S2.

### Validation of the reporter construct in Arabidopsis rosettes

The specificity of the ACC1 reporter peptide was previously tested in yeast by incubating WT and *snf1* mutant cells with fermentable glycerol and ethanol to induce SNF1 or with glucose to inhibit SNF1 activity (Deroover et al. 2016). The peptide was phosphorylated under fermenting conditions but not following glucose addition in the wild-type background, while no phosphorylation was observed in the *snf1* background, suggesting that the reporter is indeed SNF1 specific in yeast. Sanagi et al. (2021) validated an ACC1-based SnRK1 reporter in Arabidopsis mesophyll protoplasts, by showing that phosphorylation is increased by transient expression of wild-type SnRK1α1 but not by expression of a mutated form, SnRK1α1^K48A^, which is defective in the SnRK1 ATP-binding pocket (K48M). They also showed that there is less phosphorylation in protoplasts prepared from inducible *snrk1α* knockdown plants than in protoplasts prepared from wild-type plants.

We took two approaches to further validate the reporters for use in whole Arabidopsis rosettes. In the first approach, we subjected GEN and NUC lines to 3-h of extended darkness, starting at the end of the night (Supplemental Fig. S3A and Data Set S1, Experiment 1). Extended darkness is known to activate SnRK1 (see Introduction). As expected, phosphorylation of both reporters increased in extended darkness, with phosphorylation being significantly higher at ZT25 (zeitgeber time, i.e. 25 h after the previous dawn and into the extended night) and rising further at ZT27 in NUC, and being significantly higher by ZT27 in GEN. In the second approach, the NUC reporter was crossed to a 35:SnRK1α1 line that has elevated SnRK1 activity and to the 35S:SnRK1α1^K48M^ line expressing a catalytically inactive mutant protein (Jossier et al. 2009; Crozet et al. 2014; Cho et al. 2016; Supplemental Fig. S3B and Data Set S1, Experiment 2). The crossed lines and a NUC control were grown in equinoctial growth conditions for 22 d after sowing (DAS), and then harvested at ZT0, 8, 16, 24, and 28 (i.e. 4 h of extended darkness). We confirmed expression of SnRK1 proteins in these lines by immunoblotting with anti-SnRK1α1 antibody (Supplemental Fig. S3C). Phosphorylation of the NUC polypeptide was consistently higher in NUC × 35S:SnRK1α1 than in NUC × 35S:SnRK1^K48M^ (significant at ZT0, 8, 16, 24, and 28). Compared to the NUC control, reporter phosphorylation in NUC × 35S:SnRK1^K48M^ was significantly lower at ZT0, 8, 24, and 28 (with SnRK1^K48M^ apparently functioning as a dominant negative mutant protein; Cho et al. 2016), and reporter phosphorylation in NUC × 35S:SnRK1α1 was essentially control-like at ZT0, 8, 24, and 28 and significantly higher at ZT16. This experiment also confirmed the rise in reporter phosphorylation in the extended night, further validating the reporter.

### Diel changes in SnRK1 activity in equinoctial growth conditions

**Figure 1.**
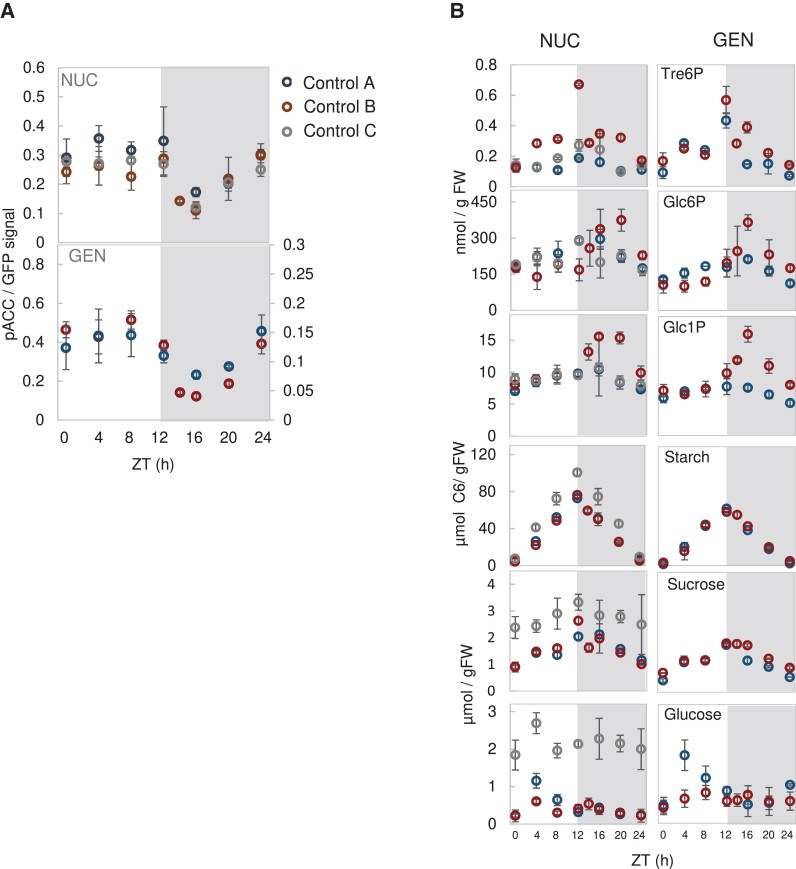
Reproducible diel response in phosphorylation of SnRK1 reporters under equinoctial growth conditions. SnRK1-reporter lines were grown in a 12-h photoperiod (irradiance 160 µmol m^−2^ s^−1^) for 22 d as controls in three independent experiments, in which additional plants were grown in parallel and subjected to different treatments. Whole rosettes were harvested at 4-h intervals through the diel cycle. A) Phosphorylation of the NUC or GEN polypeptide. This was quantified by immunoblotting as described in Materials and Methods and in Supplemental Fig. S1. B) Selected metabolites. Data are shown for the control samples from the following experiments: (i) Control A: blue symbols, NUC and GEN lines (from Experiment 3 in Supplemental Data Set S1, also shown in Fig. 2); (ii) Control B: red symbols, NUC and GEN lines (from Experiments 4 and 5 in Supplemental Data Set S1, also shown in Figs. 3 and 4; (iii) Control C: gray symbols; NUC line only (from Supplemental Data Set S1, Experiment 2, also shown in Supplemental Fig. S3B). Data are shown as mean ± SD (*n* = 3–4 biological replicates, each containing 3–5 pooled rosettes). Statistical analyses are provided separately for each time series in Figs. 2 and 3 and Supplemental Data Set S1, Experiment 2, respectively.

The NUC control in the experiment of Supplemental Fig. S3B showed a somewhat unexpected pattern, with phosphorylation remaining high until ZT8 and then falling between ZT8 and ZT16. This was unexpected as photosynthesis provides a higher C supply in the light, which would be expected to inhibit SnRK1 in the light period, while the lower C supply in the dark would be expected to stimulate SnRK1 during the night. We investigated if this unexpected diel response was robust and reproducible in two further independent experiments in which plants expressing the NUC or GEN constructs were grown in equinoctial light-dark regimes and harvested at 4-h intervals (Fig. 1A, Controls A and B). The NUC control in Supplemental Fig. S3B was further investigated by analyzing additional samples that had been harvested at ZT4, ZT12, and ZT20 (Fig. 1A, Control C). Across all these experiments, there was a robust and reproducible diel response; SnRK1 activity at dawn was maintained throughout the light period, followed by a decline after darkening to a minimum at about ZT16 and an increase in the last part of the night. The NUC and GEN lines exhibited similar responses.

We explored diel changes in metabolic status and in potential metabolite effectors of SnRK1 (Fig. 1B) in the same plant material. Obvious candidates include sugars (glucose and sucrose) and starch as indicators of C status, and sugar phosphates, such as Tre6P, Glc6P, and Glc1P, all of which were suggested previously to be directly or indirectly involved in the regulation of SnRK1 (Toroser et al. 2000; Lu et al. 2007; Zhang et al. 2009; Nunes et al. 2013b; Emanuelle et al. 2015; Broeckx et al. 2016; Hulsmans et al. 2016; Zhai et al. 2017, 2018; Ruiz-Gayosso et al. 2018). The diel responses of metabolites were reproducible and resembled previous reports (Lunn et al. 2006; Sulpice et al. 2014; Mengin et al. 2017; Flis et al. 2019; Moraes et al. 2019). In brief, starch accumulated in a near-linear manner during the light period and was mobilized in a near-linear manner at night, sucrose gradually increased in the light to a peak at ZT12 (ED) and gradually decreased during the night, and glucose levels showed a 1.5- to 3-fold transient increase at ZT4 but otherwise remained relatively low throughout the diel cycle. Tre6P changed in parallel with sucrose, as expected from previous studies (Martins et al. 2013; Figueroa et al. 2016; Peixoto et al. 2021). Glc6P and Glc1P levels remained fairly stable throughout the day, rose at the beginning of the night until ZT16 and then decreased. Starch, sucrose and glucose levels were somewhat higher in the experiment of Control C than the experiments of Controls A and B; this may reflect small shifts in the relation between C gain and growth. Variation between separately grown batches of plants has sometimes been previously observed.

These experiments did not reveal pronounced reciprocal changes in Tre6P levels and SnRK1 activity during the diel cycle, except late in the night (between ZT16 and ZT24) when Tre6P levels decreased and phosphorylation of both NUC and GEN polypeptides increased. In addition, no reciprocal changes were observed between NUC or GEN phosphorylation and sugar levels, except for sucrose in the last part of the night. However, Glc6P and Glc1P did show a reciprocal relationship with SnRK1 activity through the night; Glc6P and Glc1P initially increased after dusk (from ZT12 to ZT16), and then decreased (from ZT16 to ZT24), while phosphorylation of NUC and GEN decreased during the first part of the night and then increased (see later for more analysis).

### Perturbations of the light regime

To further explore how SnRK1 is regulated by changes in metabolites, by light and/or by endogenous (e.g. circadian clock) rhythms, we investigated NUC and GEN phosphorylation during perturbations of the light regime.

**Figure 2.**
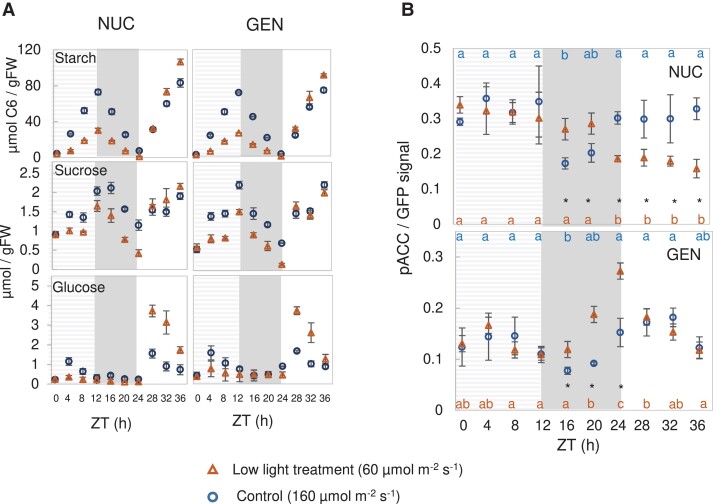
SnRK1 activity in Arabidopsis plants transferred to low irradiance for one light period. NUC and GEN lines were grown in a 12-h photoperiod at 160 µmol m^−2^ s^−1^ irradiance for 19 d. At 20 DAS, starting at dawn (ZT0), irradiance was reduced to 60 µmol m^−2^ s^−1^ for the following 12 h (treatment light period), plants were darkened at ZT12 (treatment night), and at ZT24 were re-illuminated at an irradiance of 160 µmol m^−2^ s^−1^ (recovery day), as described in Moraes et al. (2019). Samples from both genotypes and from the corresponding control treatment (plants left in the original growth light regime; corresponds to Control A in Fig. 1) were harvested at 4-h intervals from ZT0-ZT36 (with respect to dawn on the treatment day). A) Soluble sugars and starch. B) Phosphorylation of NUC (top) and GEN (bottom) SnRK1-reporter polypeptides. The dashed gray background denotes the low-light treatment day. Results are shown as mean ± SD (*n* = 3–4 biological replicates). Statistical analysis: letters indicate significant (*P* < 0.05) changes between different times for a given genotype and asterisks indicate significant (*P* < 0.05) differences between lines at a given time point, according to one-way ANOVA and pairwise multiple comparison post testing using the Holm–Sidak method. The original and additional data are available in Supplemental Data Set S1, Experiment 3.

The first treatment was based on a perturbation described in Moraes et al. (2019). Plants were grown in equinoctial conditions under 160 µmol m^−2^ s^−1^ irradiance for 19 d, and then transferred to 60 µmol m^−2^ s^−1^ for one light period (treatment light period), darkened at ZT12 at the same time as in the initial growth conditions (treatment night), and then returned to 160 µmol m^−2^ s^−1^ on the next day (recovery light period; Fig. 2). Control plants were left in the original growth conditions (this is Control A from Fig. 1, and is replotted in Fig. 2 to allow direct comparison with the low-light treatment). The response of starch and sugars closely resembled that in Moraes et al. (2019). Compared with control plants, a single low-irradiance day led to slower starch accumulation and a decrease in the levels of sucrose and glucose during the treatment light period, and slower starch mobilization and lower sugar levels in the following night (Fig. 2A; Supplemental Data Set S1, Experiment 3). During the recovery light period, glucose was higher and starch accumulation was faster than in the light period in control plants, indicating that growth was restricted (see also Gibon et al. 2004; Sulpice et al. 2014; Mengin et al. 2017).

Phosphorylation of NUC and GEN polypeptides (Fig. 2B) showed a combination of expected and unexpected features, which were partially shared between the NUC and GEN lines but also showed some differences. Rather surprisingly, a sudden decrease in irradiance had no immediate impact on NUC or GEN phosphorylation in the treatment light period. However, it did have significant effects in the following night and during the recovery day. For NUC, there was no initial drop in phosphorylation after darkening, with the result that phosphorylation was significantly higher than in the control (where it decreased in the first part of the night). However, after ZT20, there was a sharp decrease in NUC phosphorylation and this persisted during the recovery light period. For GEN, phosphorylation increased significantly compared with the control by ZT16, and increased even further as the night progressed, followed by a decrease to control levels in the recovery light period.

It is rather surprising that, after a shift to low light, the decrease of sugar levels was not accompanied by an increase in NUC or GEN phosphorylation in the light period. This indicates that at this time in the diel cycle, nuclear and extra-nuclear SnRK1 activity are not responding to current C availability as reflected by overall sugar levels. At night, NUC and GEN phosphorylation were higher in plants exposed to low irradiance during the preceding light period than in the control, reflecting the lower C availability, but this response was much more marked for GEN than NUC. Indeed, NUC signal fell at the end of the night, even though sugars remained low. In the recovery day, when sugars were higher than in the control, NUC phosphorylation was lower than the control as would be expected if it were responding to current C availability. However, GEN phosphorylation was high, indicating that extra-nuclear SnRK1 activity was not responding to current C availability. Indeed, considering that GEN is a composite of nuclear and of cytoplasmic activity, it is likely that cytoplasmic SnRK1 activity was actually increased since NUC signal activity was decreased at this time (see above).

The second treatment was based on an experiment reported by Fernandez et al. (2017), in which plants were grown in equinoctial conditions with 160 µmol m^−2^ s^−1^ irradiance for 19 d and then transferred to continuous lower irradiance. The original study of Fernandez et al. (2017) investigated starch degradation in the light, and the low irradiance was used to avoid overaccumulation of starch in continuous light. The rationale for the current experiment was to test whether the diel changes in phosphorylation activity of SnRK1 are driven by the external light regime or by endogenous cues.

**Figure 3.**
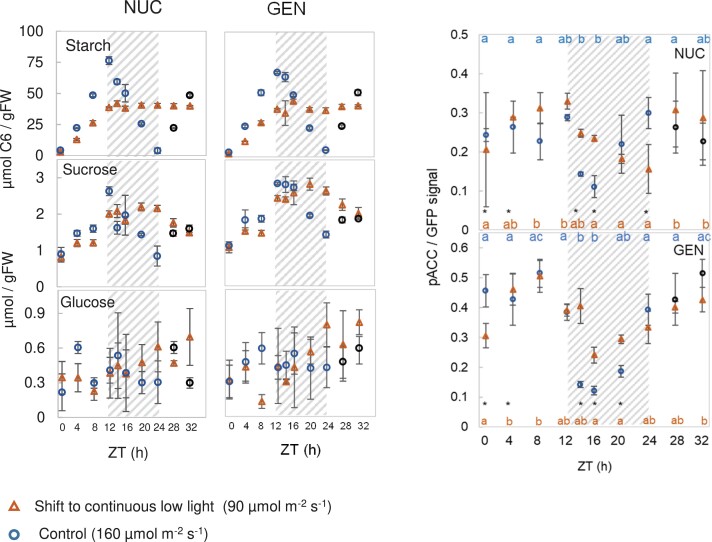
SnRK1 activity in Arabidopsis plants transferred to continuous low light. NUC and GEN lines were grown in a 12-h photoperiod at 160 µmol m^−2^ s^−1^ irradiance for 19 d. At 20 DAS, plants were shifted at dawn (ZT0) to continuous low light (90 µmol m^−2^ s^−1^) for 32 h, while corresponding controls were kept in the original growth conditions (12-h photoperiod, 160 µmol m^−2^ s^−1^; corresponds to Control B in Fig. 1). A) Diel changes of sugars and starch. B) Phosphorylation of NUC (top) and GEN (bottom) SnRK1-reporter polypeptides. The dashed gray background represents the night for control samples and subjective night for treated samples. Data points for control samples at ZT28 and ZT32 were not measured and the plotted values are replicated from those at ZT4 and ZT8, respectively (black circles). Results are shown as mean ± SD (*n* = 3 biological replicates). Statistical analysis: letters indicate significant (*P* < 0.05) changes between different times for a given treatment and asterisks represent significant (*P* < 0.05) differences between the low-light treatment and the control at a given time point according to one-way ANOVA with pairwise multiple comparison post testing using the Holm–Sidak method. The original and additional data are available in Supplemental Data Set S1, Experiment 4.

We grew plants in standard equinoctial conditions at 160 µmol m^−2^ s^−1^, then transferred them, at dawn on Day 20 after sowing, to continuous light with an irradiance of 90 µmol m^−2^ s^−1^, and sampled them at 4-h intervals for 32 h (Fig. 3). Control plants were left in the original growth conditions (this corresponds to Control B from Fig. 1, and is replotted in Fig. 3 to allow direct comparison with the continuous low-light treatment). After transfer to continuous low light, starch accumulated for about 12 h, although more slowly than in control plants, and starch levels then plateaued or even declined slightly in the subjective night from ZT12 onwards (Fig. 3A; Supplemental Data Set S1, Experiment 4). Sugar levels were lower than in the control in the subjective day period (ZT0–ZT12) but rose to reach higher levels from ZT16 onwards. These changes of starch and sucrose reflect a gradual but progressive increase in the rate of starch mobilization from about ZT10 onwards (see Fernandez et al. 2017; Ishihara et al. 2022 for details). Compared with the control, Glc6P was only marginally higher in the subjective day, and was comparable (NUC) or slightly lower (GEN) in the subjective night (Supplemental Fig. S4). Once again, we observed that lower sugar levels during the first 12 h of the 24-h cycle were not accompanied by a significant increase in phosphorylation of NUC or GEN, which essentially exhibited a control-like response until ZT12 (Fig. 3; Supplemental Data Set S1, Experiment 4). In both lines, SnRK1 activity in continuous low light was higher in the subjective night than in the control during the night. Nevertheless, the decline in SnRK1 activity that is observed after darkening control plants at ZT12 was also observed under low continuous light, although in a weakened form. NUC phosphorylation showed a gradual significant decrease (relative to ZT12) from ZT14 to ZT24, while GEN phosphorylation started to decrease by ZT12 and declined until ZT16 (Fig. 3B). In the next subjective day, phosphorylation levels of both NUC and GEN resembled those in the control.

These observations confirmed that a fall in C availability due to decreased irradiance does not lead to increased SnRK1 activity in the first part of the 24-h cycle. They also point to an endogenous component contributing to the decline in SnRK1 activity from about ZT12 onwards. Furthermore, we noted a reciprocal relationship between diel changes in Glc6P and SnRK1 activity, particularly with NUC phosphorylation.

### Response in a T28 cycle

The Arabidopsis circadian clock is mainly entrained by light at dawn and imposes an endogenous 24-h rhythmicity even when the external cycle deviates from 24 h (Millar et al. 1995; Locke et al. 2005; Graf et al. 2010). Growing plants in a light-dark cycle that deviates from 24 h is therefore a good way to search for oscillatory responses that are driven directly or indirectly by the clock.

**Figure 4.**
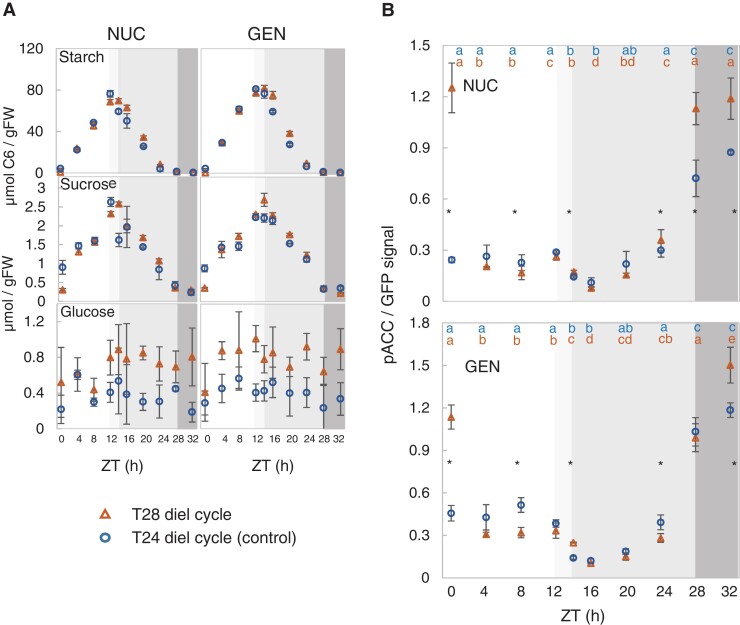
SnRK1 activity in Arabidopsis plants grown in T24 and T28 light-dark cycles. NUC and GEN lines were grown from sowing in a 14-h light/14 h dark (T28) cycle under 160 µmol m^−2^ s^−1^ irradiance for 19 d, in parallel with control plants that were grown in a 12-h light/12-h dark (T24) cycle. At 20 DAS (as defined by the T24 cycle), starting at dawn, plants were harvested at 4-h intervals throughout the light-dark cycle (open symbols). At the end of the light-dark cycle, additional plants were left in darkness and harvested for two further time points for the plants grown in a T24 cycle (i.e. at ZT28 and ZT32), and for one further time point for plants grown in a T28 cycle (i.e. at ZT32). This experiment was carried out in parallel with that shown in Fig. 3. The T24 data from ZT0 to ZT24 are identical to the control treatment in the continuous low-light treatment of Fig. 3 (Control B), with additional time points added for plants that were left in the dark from ZT24 onwards and harvested at ZT28 and ZT32. The vertical dotted line indicates the time at which control plants were transferred to continued darkness. A) Soluble sugars and starch. B) Phosphorylation of NUC (top) and GEN (bottom) SnRK1-reporter polypeptides. The white background represents light in both T cycles and the gray-shaded areas represent darkness (ZT12–ZT24 for the T24 cycle and ZT14–ZT28 for the T28 cycle) in one or both T cycles: pale gray denotes darkness in the T24 and light in the T28 cycle, mid-gray denotes darkness in both T-cycles, and dark gray represents extended night in both T cycles. Black closed symbols represent time points in extended darkness. Results are shown as mean ± SD (*n* = 3 biological replicates). Statistical analysis: letters indicate significant (*P* < 0.05) changes between different times for a given genotype and asterisks represent significant (*P* < 0.05) differences between T28 versus T24 at a given time point according to a one-way ANOVA with pairwise multiple comparison post testing using the Holm–Sidak method. The original and additional data are available in Supplemental Data Set S1, Experiment 5.

We grew plants in a 14-h light/14-h dark (T28) or a standard 12-h light/12-h dark (T24) cycle for 19 d with 160 µmol m^−2^ s^−1^ irradiance and harvested them at 4-h intervals on Day 20 after sowing, including a 4- or 8-h period of extended darkness for the T28 and T24 control, respectively (Fig. 4). This experiment was carried out in parallel with the experiment of Fig. 3, in which plants were transferred to constant 90 µmol m^−2^ s^−1^ irradiance, and shares the same control (Control B). As previously seen (Graf et al. 2010), in a T28 cycle starch is exhausted at about ZT24, resulting in transient C starvation and low sucrose levels from ZT24 to ZT28 (i.e. the four hours preceding the next dawn; Fig. 4A; Supplemental Data Set S1, Experiment 5) as well as lower Glc6P levels through the diel cycle (Supplemental Fig. S5A). Correspondingly, phosphorylation of NUC and GEN at ZT0 (dawn) was almost 6- and 3-fold higher in the T28 cycle than the T24 cycle, comparable with the increase observed after extending the night in a T24 cycle (Fig. 4B; Supplemental Data Set S1, Experiment 5, see also Supplemental Fig. S3A). After illumination, NUC and GEN phosphorylation decreased strongly in a T28 cycle, falling to levels that were lower than in a T24 cycle for most of the remaining light-dark cycle (significant for both NUC and GEN at ZT8, ZT14, and ZT24). Importantly, NUC and GEN phosphorylation decreased between ZT12 and ZT14 in a similar manner in both the T28 and T24 cycle plants, even though the former were still in the light while the latter were already in the dark. Together with the response in continuous light (see above), these observations point to a component with 24-h rhythmicity contributing to the diel regulation of SnRK1 activity.

### Manipulation of Tre6P levels

Tre6P is a signal of sucrose levels (Lunn et al. 2006, 2014; Figueroa and Lunn 2016; Fichtner and Lunn 2021) and is widely accepted as an inhibitor of SnRK1, at least in vitro (Zhang et al. 2009; Zhai et al. 2018). However, the evidence that these interactions occur in vivo is largely correlative (Martínez-Barajas et al. 2011; Wingler et al. 2012; Nunes et al. 2013a; Henry et al. 2015; Nuccio et al. 2015; Griffiths et al. 2016; Peixoto et al. 2021; Zacharaki et al. 2022). We exploited our SnRK1-reporter lines to develop a genetic approach to test whether Tre6P inhibits SnRK1 activity in vivo. We have previously used inducible-TPS (iTPS) lines, expressing the *Escherichia coli* TPS under the control of the *Aspergillus nidulans* ethanol-inducible ALCOHOL DEHYDROGENASE REGULATOR (alcR) and alcA promoter to investigate the impact of a short-term induced increase in Tre6P on starch, organic acid and nitrogen metabolism (Martins et al. 2013; Figueroa et al. 2016). We now introgressed the NUC and GEN reporters into a well characterized iTPS line (TPS29.2; Martins et al. 2013; Figueroa et al. 2016) and into its corresponding empty-vector control, alcR. Four homozygous lines were generated: two lines containing the alcR gene and the inducible *TPS* (NUC × iTPS #6.3 or GEN × iTPS #2.4), and two control lines with only the alcR gene (NUC × alcR #1.2 or GEN × alcR #1.1).

These lines were grown in long-day conditions (16-h photoperiod, 160 µmol m^−2^ s^−1^ irradiance). On Day 21 after sowing, plants were sprayed at ZT1 with either water (control) or 2% (v/v) ethanol to induce the expression of the bacterial TPS (Supplemental Fig. S6). Two whole rosettes were harvested and pooled at 4, 6, and 8 h after spraying. Transient expression of the bacterial TPS protein was detectable within 4 h of induction (Supplemental Fig. S6B), consistent with previous experiments with the parental iTPS line (Martins et al. 2013; Figueroa et al. 2016). After ethanol induction, there was a strong and consistent decrease in SnRK1 nuclear activity (in NUC × iTPS) and a small decrease in overall SnRK1 activity after 4 and 6 but not after 10 h in GEN × iTPS (Supplemental Fig. S6A and Data Set S1, Experiment 6). This led us to focus on the NUC × iTPS line (#6.3) in subsequent experiments. There was no consistent effect of ethanol on NUC or GEN phosphorylation in the control alcR lines, in which alcR is expressed but there is no bacterial *TPS* gene to be expressed under the control of the alcA promoter. This control excludes any risk of off-target effects in the presence of ethanol or due to expression of alcR (Randall 2021).

**Figure 5.**
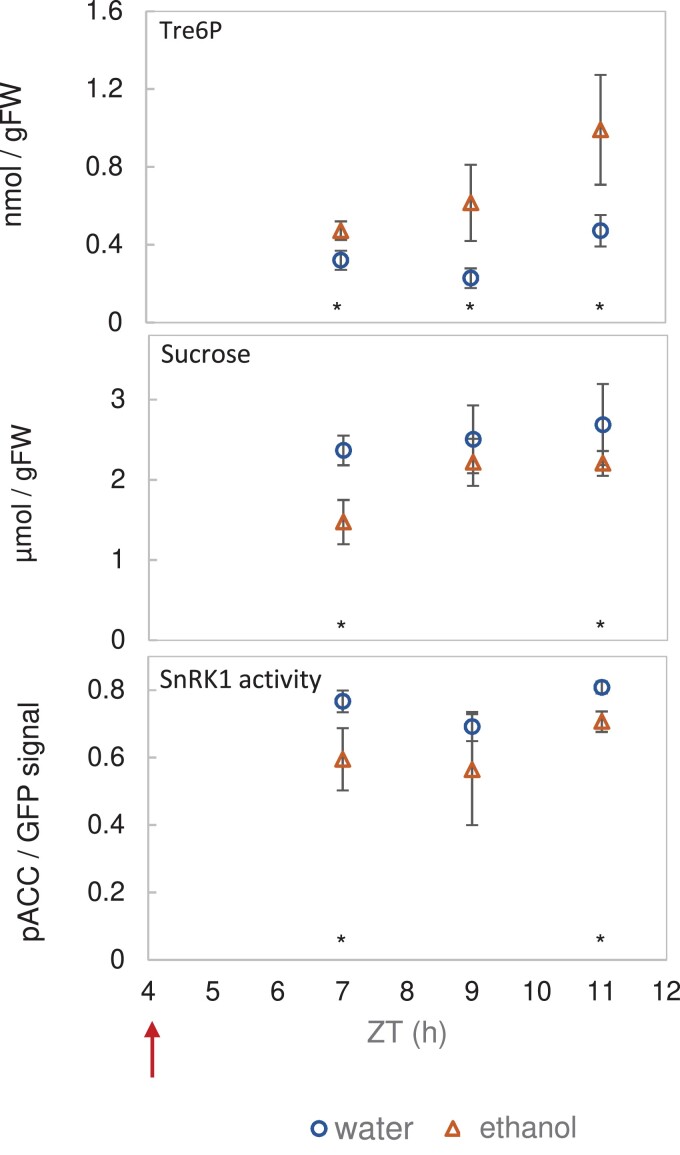
Elevated Tre6P in the light period inhibits nuclear SnRK1 activity. Line #6.3 (NUC × iTPS) was grown in a 12-h photoperiod at 160 µmol m^−2^ s^−1^ irradiance for 20 d. At 21 DAS, plants were sprayed at ZT4 (arrow) with 2% (v/v) ethanol to induce the expression of bacterial TPS (otsA) or with water as a mock-induction control, and then harvested at ZT7, ZT9, and ZT11 for measurement of Tre6P (top panel), sucrose (middle), and the phosphorylation status of the NUC polypeptide (bottom) as a readout of nuclear SnRK1 activity in vivo. Results are shown as mean ± SD (*n* = 3–4 biological replicates). Statistical analysis: asterisks indicate significant (*P* < 0.05) differences between the ethanol-induced plants and water controls according to a one-way ANOVA with pairwise multiple comparison post testing using the Holm–Sidak method. Additional metabolites are shown in Supplemental Fig. S7. The original data are available in Supplemental Data Set S1, Experiment 7.

To confirm the inhibitory effect of Tre6P induction in the light period, we conducted a more extensive experiment. Line #6.3 was grown in equinoctial conditions under 160 µmol m^−2^ s^−1^ irradiance for 20 d (the standard conditions of the experiments of Figs. 1–4), sprayed on 21 DAS with either water or 2% (v/v) ethanol at ZT4, and triplicate samples harvested at ZT7, ZT9, and ZT11 and separately analyzed (Fig. 5; Supplemental Data Set S1, Experiment 7). Ethanol spraying led to a significant 41%, 169%, and 130% increase in Tre6P levels at ZT7, ZT9, and ZT11, compared with control plants (Fig. 5). Starch accumulation was marginally enhanced, sucrose levels decreased at ZT7 and ZT11 (Fig. 5), reducing sugars were not consistently or significantly affected, and the levels of many organic acids increased (Supplemental Fig. S7 and Data Set S1, Experiment 7), as previously reported by Figueroa et al. (2016). NUC phosphorylation showed a significant but small decrease (12%–23%) following ethanol spraying, compared with the water-sprayed control (Fig. 5). This provides evidence that Tre6P indeed inhibits nuclear SnRK1 activity in vivo, but the impact seems to be rather small at the whole-rosette level.

**Figure 6.**
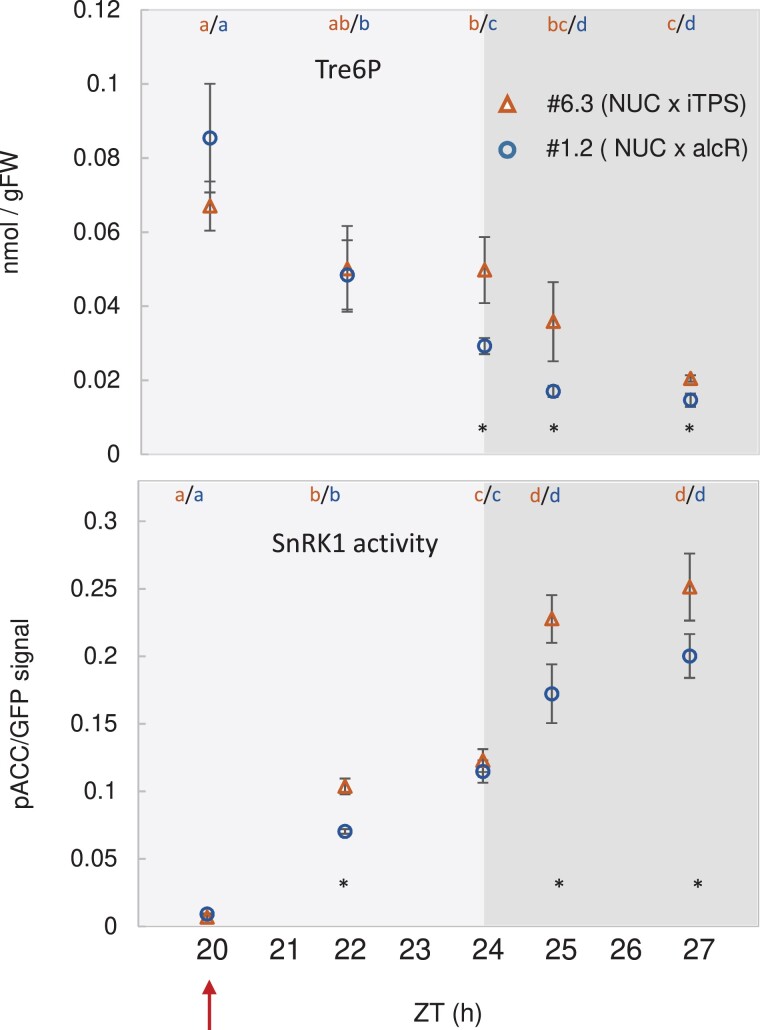
Transiently high Tre6P at the end of the night leads to an increase in nuclear SnRK1 activity. Line #6.3 (NUC × iTPS) and #1.2 control (NUC × alcR) were grown in short-day conditions (6 h light/18 h dark) at 160 µmol m^−2^ s^−1^ irradiance for 25 d. At 26 DAS, plants were sprayed with 2% (v/v) ethanol at ZT20 (arrow) to induce expression of bacterial TPS (otsA). Rosettes were harvested at ZT20, ZT22, and ZT24 (end of normal night), and at ZT25 and ZT27 (extended darkness) for measurement of: Tre6P (top panel) and the phosphorylation of the NUC polypeptide (bottom) as a readout of nuclear SnRK1 activity in vivo. Results are shown as mean ± SD (*n* = 3–4 biological replicates). Statistical analysis: letters indicate significant (*P* < 0.05) changes between different times for a given genotype and asterisks indicate significant (*P* < 0.05) differences between the #6.3 (NUC × iTPS) and #1.2 control (NUC × alcR) lines according to a one-way ANOVA with pairwise multiple comparison post testing using the Holm–Sidak method. Additional metabolites are shown in Supplemental Fig. S8. The original data are available in Supplemental Data Set S1, Experiment 8.

We hypothesized that if Tre6P were not the only regulator of SnRK1, other factors might counteract, and thereby partly mask, the effect of Tre6P. Therefore, we carried out a further experiment in which TPS was induced in conditions when sugars and other metabolites were low, and might not mask the expected inhibition by Tre6P. Plants were grown in short-day conditions (6 h light/18 h dark) for 21 DAS with 160 µmol m^−2^ s^−1^ irradiance, sprayed with either water or 2% (v/v) ethanol toward the end of the night (ZT20) and harvested at ZT20, ZT22, ZT24, ZT25, and ZT27 (Fig. 6). We included an alcR control line, and all comparisons were made between ethanol-induced NUC × iTPS (#6.3) and ethanol-induced NUC × alcR #1.2 as the control. Under these conditions, starch was almost completely exhausted and soluble sugars were at low levels at the end of the night (Supplemental Fig. S8A). Tre6P levels declined strongly between ZT20 and ZT27 in the control. Induction of TPS slowed down this decline (Fig. 6), with Tre6P levels at ZT24, ZT25, and ZT27 being significantly higher in ethanol-sprayed NUC × iTPS #6.3 than in the NUC × alcR (#1.2) control at the same time (by 59%, 112%, and 33%, respectively). Phosphorylation of NUC in the control rose between ZT20 and ZT24, and rose further at ZT25 and ZT27 (Fig. 6). This resembles the response of wild-type plants at the end of the night and in an extended night (see above). Unexpectedly, induction of TPS did not attenuate but instead promoted this increase in nuclear SnRK1 activity. Thus, the inhibitory effect of elevated Tre6P appears to be context-dependent.

The increase in Tre6P levels after ethanol spraying in the night was accompanied by a slight slowing of starch breakdown and lower glucose levels (Supplemental Fig. S8A). This confirms the response previously reported in Martins et al. (2013), but in the present case for induction at a later time in the night. The increase in Tre6P levels was also accompanied by increases in malate, pyruvate and glycerol 3-phosphate (Gly3P) and a decrease in phospho*enol*pyruvate (PEP) levels (Supplemental Fig. S8B and Data Set S1, Experiment 8), indicating that the stimulation by Tre6P of anaplerotic flux that was previously reported in the light (Figueroa et al. 2016) may also occur at the end of the night. Some of these downstream responses to elevated Tre6P may lead to the unexpected increase in nuclear SnRK1 activity after iTPS induction.

Overall, these results show that Tre6P is not the sole regulator of SnRK1, and its inhibitory effect appears to be context dependent. Indeed, in some conditions, secondary changes caused by Tre6P induction may lead, indirectly, to a stronger positive effect on SnRK1 activity than the direct inhibitory effect of elevated Tre6P itself.

### Correlation analysis of relationships between SnRK1 activity and metabolite levels

Taken together, our experiments point to complex and somewhat flexible regulation of SnRK1 activity in conditions where C status is changing in a range above that prevailing during C starvation. We therefore investigated more systematically the relationship between diel changes of metabolite levels and NUC or GEN phosphorylation.

We did this first for the three control NUC (Controls A, B, and C) and the two control GEN (Controls A and B) data sets in undisturbed equinoctial diel cycles (see Fig. 1 for plots of the time courses of SnRK1 activity and metabolite levels). We performed regression analysis separately for each experiment on all biological replicates and time points across the whole diel cycle (Table 1, for regression plots see Supplemental Data Set S2). Tre6P levels were not correlated with either NUC (*R*^2^ = 0.007, 0.004, and 0.004) or GEN phosphorylation (*R*^2^ = 0.001 and 0.046). Glc6P levels were not consistently related to NUC phosphorylation with *R*^2^ values of 0.048, 0.21 (negative and significant, *P* = 0.022), and 0.02 but were negatively correlated with GEN phosphorylation with *R*^2^ values of 0.22 (*P* = 0.03) and 0.58 (*P* = 2.24 × 10^−5^). Similar relationships were observed for Glc1P which, due to the near-equilibrium reaction catalyzed by phosphoglucomutase, can be expected to change in parallel with Glc6P. Starch was not correlated with either NUC or GEN phosphorylation. Sucrose was not correlated with NUC and was negatively correlated with GEN phosphorylation in one but not the other experiment. Glucose was not correlated with NUC, and positively correlated with GEN phosphorylation in one but not the other experiment.

**Table 1.** Correlations between metabolite levels and NUC or GEN phosphorylation in equinoctial diel cycles

Control treatment	Metabolite	NUC			GEN		
		*R* ^2^	*P*-value	±	*R* ^2^	*P*-value	±
A	Tre6P	0.007	0.673	+	0.001	0.965	+
	Glc6P	0.048	0.270	−	0.225	0.030	−
	Glc1P	0.056	0.236	−	0.036	0.412	−
	Starch	0.006	0.706	+	0.051	0.324	−
	Sucrose	0.085	0.139	−	0.058	0.295	−
	Glucose	0.121	0.076	+	0.272	0.015	+
B	Tre6P	0.004	0.778	+	0.046	0.325	−
	Glc6P	0.215	0.022	−	0.583	2.24 × 10^−5^	−
	Glc1P	0.418	0.001	−	0.683	1.17 × 10^−6^	−
	Starch	0.081	0.190	−	0.081	0.189	−
	Sucrose	1 × 10^−8^	0.685	−	0.324	0.005	−
	Glucose	0.054	0.687	−	2 × 10^−5^	0.985	−
C	Tre6P	0.004	0.233	−	ND	ND	
	Glc6P	0.002	0.766	−	ND	ND	
	Glc1P	0.02	0.373	+	ND	ND	
	Starch	0.032	0.364	−	ND	ND	
	Sucrose	0.007	0.678	+	ND	ND	
	Glucose	0.013	0.561	−	ND	ND	

Correlation analysis was performed separately on the three independent NUC and the two independent GEN control experiments that are compiled in Fig. 1. The analysis was performed with the “least squares” method, using individual replicates for the complete diel cycle (ZT0 to ZT24). The column ± provides information about the slope of the regression: positive (+), negative (−). Control treatments A and B correspond to the diel controls shown in Fig. 2 (Experiment 3 in Supplemental Data Set S1) and Figs. 3 and 4 (Experiments 4 and 5 in Supplemental Data Set S1). The experiment corresponding to treatment C (Experiment 2 in Supplemental Data Set S1) was performed with the NUC line only. ND, not determined. Individual plots are provided in Supplemental Data Set S2.

We also performed regression analysis separately on the light period (ZT4–ZT12) and night (ZT16–ZT24) data sets (Supplemental Table S1). There were almost no significant relationships in the light period. This can be expected as even though the levels of most sugars, sugar phosphates, and many other metabolites change, SnRK1 activity is rather constant during the light period. During the night there were many negative relationships between NUC or GEN phosphorylation and metabolite levels, including Tre6P, Glc6P, Glc1P, starch, and sucrose. These negative relationships reflect falling levels of many metabolites toward the end of the night, as starch is gradually exhausted. They may individually or collectively contribute to the rise in NUC and GEN activity at this time.

Pairwise correlation analyses between metabolites (Supplemental Table S2) revealed that most of the measured metabolites are positively correlated with each other. This reflects the highly coordinated nature of the changes in metabolism in diel cycles, and underlines that some of the observed correlations between metabolites and NUC or GEN phosphorylation may be secondary.

The diel changes in SnRK1 activity and metabolite levels included abrupt changes after illumination or darkening. These may not be well represented in correlations based on changes across the entire cycle. To place more weight on rapid transient changes, we calculated the difference between the average absolute levels at consecutive time points in the diel cycle and divided this by the time difference to obtain the direction and rate of change of a given trait in a given time interval. Compared with absolute values, derivative values provide more information about changes in the balance in metabolism. The derivative values were used for linear regression analyses as described above, except that analyses were only performed for the entire diel cycle (Supplemental Table S3 and Data Set S2). Changes in both NUC and GEN phosphorylation are significantly and negatively correlated to changes in Glc6P and Glc1P, with changes in Glc6P showing the strongest reciprocal agreement with changes in GEN (highest *R*^2^ and lowest *P*-values).

We also performed correlation analyses for treatments where diel metabolism was perturbed by transferring plants to continuous light or by growing plants in T28 cycles, or where Tre6P levels were elevated by ethanol spraying of iTPS lines (for details, see Supplemental Text S1, and for analyses, see Supplemental Tables S4, S5 and Data Set S2). These analyses detected significant correlations between Tre6P, Glc6P and Glc1P, and NUC (*R*^2^ = 0.46, 0.41, and 0.23; *P* = 2.6 × 10^−4^, 0.001, and 0.018, respectively) or GEN phosphorylation (*R*^2^ = 0.34, 0.31, 0.26; *P* = 0.004, 0.005, and 0.013, respectively) after transfer to continuous low light. There were significant correlations between Glc6P and NUC (*R*^2^ = 0.89; *P* = 1.9 × 10^−13^) phosphorylation activity in a T28 cycle. In experiments in which Tre6P was induced in the light period, NUC phosphorylation correlated negatively with Tre6P in the control water-sprayed plants (*R*^2^ = 0.67; *P* = 0.004) but not when ethanol was sprayed to increase Tre6P (*R*^2^ = 0.12; *P* = 0.76; Supplemental Table S6). This observation is consistent with the idea that SnRK1 activity is regulated by a network including Tre6P and that this network adjusts to decrease the sensitivity to Tre6P when Tre6P levels are artificially elevated.

## Discussion

### Monitoring in vivo SnRK1 activity in plants by expression of a chimeric protein reporter

Two approaches have been widely utilized to study the regulation of SnRK1 activity in plants. One is in vitro phosphorylation assays using a peptide with a conserved SNF1/SnRK1/AMPK recognition sequence (Dale et al. 1995). in vitro studies are useful for identifying potential ligands and regulators, including interacting proteins, and can reveal changes in maximum catalytic activity of SnRK1. However, removal of the enzyme from its in vivo context means that activity measurements on in vitro extracts provide only a limited information about activity in vivo. The other approach is to monitor in vivo readouts of SnRK1 activity, such as changes in the abundance of marker gene transcripts (Baena-González et al. 2007; Zhang et al. 2009; Ramon et al. 2019; Crepin and Rolland 2019). However, while relatively easy to quantify, transcripts are only indirect readouts of SnRK1 activity. Their abundance reflects changes in the activity of transcriptional regulators that are the direct targets for phosphorylation by SnRK1, but can also be influenced by SnRK1-independent changes in transcriptional or post-transcriptional regulation. The ACC1-based polypeptide reporters developed by Deroover et al. (2016) have been used to monitor in vivo SnRK1 activities in Arabidopsis (Muralidhara et al. 2021; Sanagi et al. 2021; Henninger et al. 2022; Belda-Palazón et al. 2022), and we applied this approach to study in vivo SnRK1 activity during the diel cycle in Arabidopsis rosettes. This approach does have some potential limitations. The ACC1-based polypeptide might be phosphorylated by other protein kinases in addition to SnRK1, or the extent of phosphorylation may be affected by changes in the activity of endogenous protein phosphatases. Further, the approach detects phosphorylation of a heterologous polypeptide, and so might not capture modifications of SnRK1 function that alter its ability to recognize different endogenous protein targets. In addition to the tests performed in leaf mesophyll protoplasts by Sanagi et al. (2021), we validated our approach by confirming that phosphorylation of the reporter increased when SnRK1 activity in planta was enhanced by extended darkness (Supplemental Fig. S3A) or overexpression of SnRK1α1, and that phosphorylation of the reporter decreased when SnRK1 activity was suppressed by expression of a dominant negative form (35S:SnRK1α1^K48M^; Supplemental Fig. S3B). Nevertheless, the potential complications noted above should be borne in mind when interpreting the results.

SnRK1 protein is located in the nucleus and in various extra-nuclear locations, and probably has different targets in each of these cellular locations (see Introduction and Crozet et al. 2014; Ruiz-Gayosso et al. 2018; Ramon et al. 2019; Baena-González and Lunn 2020). We introduced GFP-tagged reporter constructs with a nuclear localization sequence (NUC) and without a nuclear localization sequence (GEN), and confirmed that the former is localized to the nucleus, whereas the latter has a more general location inside and outside the nucleus (Supplemental Fig. S2). In many treatments, including unperturbed diel cycles (Fig. 1) and after transfer to extended darkness (Supplemental Fig. S3A), the phosphorylation state of the NUC and GEN peptides changed in a qualitatively similar manner. However, in some perturbations they changed in a dissimilar or even opposite manner (see Figs. 2 and 3), presumably reflecting differing or even opposing changes in SnR, K1 activity inside and outside the nucleus, which might be due to differential regulation or to altered subcellular distribution of SnRK1.

### Diel changes in SnRK1 activity point to it operating in a network that buffers C status

It is well known that SnRK1 activity increases in stress and starvation conditions (Baena-González et al. 2007; Sheen and Baena-Gonzalez 2008; Mair et al. 2015; Cho et al. 2016; Pedrotti et al. 2018; Ramon et al. 2019). However, it has emerged that SnRK1 also plays a role in benign conditions (Ramon et al. 2019; Baena-González and Lunn 2020; Peixoto et al. 2021). After establishing that our in vivo assay captures the expected increase in SnRK1 activity in C-depleted conditions, we focused on monitoring SnRK1 activity in conditions where C availability is changing but without stressful extremes of C starvation or surplus. To do this, we grew Arabidopsis in a 12-h photoperiod with 160 µmol m^−2^ s^−1^ irradiance as a standard condition in which growth is restricted by the C supply, but not strongly (Sulpice et al. 2014).

One major finding was that in vivo SnRK1 activity was relatively high in the light period, fell after ZT12 (coinciding with darkening in our standard growth conditions) to a minimum at ZT16 and then rose until the end of the night (Fig. 1). Activity during the unperturbed diel cycle was several times lower than in extended darkness or other treatments that led to C depletion (see Figs. 4 and 6, Supplemental Fig. S3A). Nevertheless, it was unexpected to observe higher SnRK1 activity in the light period than during the first part of the night, because photosynthesis provides far more C in the light than can be provided by mobilizing C reserves like starch in the night. Plants face a daily challenge to balance the utilization of newly fixed C for immediate growth with the need to lay down C reserves (e.g. starch) and to use these prudently to survive through the night. Therefore, plants must carefully regulate the rate of starch accumulation in the light period, the rate of starch mobilization at night, and the rate at which C is used for growth at different times in the diel cycle (Ishihara et al. 2015a; Ivakov et al. 2017; Mengin et al. 2017). The diel pattern of SnRK1 activity that we observed presumably reflects coordinated regulation of C utilization for reserve formation and growth throughout the diel cycle. Incidentally, the relatively low SnRK1 activity during the night adds to the evidence that C homeostasis is maintained across the diel light-dark cycle. In agreement, sucrose levels remained relatively high for much of the night.

A second unexpected finding was that SnRK1 activity did not increase in response to a decrease in overall C availability in the light. Illumination from dawn onwards at a lower irradiance than growth irradiance did not lead to an increase in the phosphorylation status of either the NUC or GEN reporter polypeptides, compared with that at growth irradiance (Figs. 2 and 3). Although photosynthesis was not measured in these experiments, the low irradiance treatments did lead to a consistent decrease in the levels of sucrose and reducing sugars (Figs. 2B and 3B; Supplemental Data Set S1, Experiments 3 and 4; see also Moraes et al. 2019). Further, these changes in irradiance occurred in the range where Arabidopsis photosynthesis is light limited (see Borghi et al. 2019), and previous studies have shown that similar drops in irradiance lead to a large decrease in photosynthetic rate (see Fernandez et al. 2017; Moraes et al. 2019). On the other hand, treatments that led to a decreased dusk starch content and slower rate of starch mobilization during the night did lead to a consistent increase in vivo SnRK1 activity in the first part of the night.

These observations point to SnRK1 being regulated in a more flexible manner in the light than in the dark. This flexibility is also revealed by the response when plants are transferred to low irradiance for one light period and then returned to growth irradiance. This perturbation led to higher levels of sugars and faster accumulation of starch when plants were returned to growth irradiance, compared with control plants that had been left at growth irradiance (Fig. 2B). These observations indicate that low irradiance results in a restriction of growth on the following day after returning plants to previous growth irradiance (see also Moraes et al. 2019). SnRK1 activity showed a complex pattern after returning to the previous growth irradiance (Fig. 2); phosphorylation of NUC was lower than in control plants, reflecting the higher levels of sugars, whereas phosphorylation of GEN resembled that in control plants implying that extra-nuclear SnRK1 activity was higher than in control plants despite sugar levels also being higher. It is known that growth in the light period is restricted when Arabidopsis experiences a shortfall in C in the preceding 24-h cycle (Gibon et al. 2004; Sulpice et al. 2014; Flis et al. 2016; Mengin et al. 2017; Moraes et al. 2019). Our observations indicate that increased extra-nuclear SnRK1 activity might contribute to this restriction of growth and the resulting increase in starch accumulation.

There may also be more ways for the plant to cope with a lower rate of photosynthesis in the light than there are to adjust to a shortfall of starch at night. For example, the impact of decreased irradiance on Glc6P levels in the light period was less marked than the impact on sugar levels (Supplemental Fig. S4, see also Moraes et al. 2019). This may reflect compensatory responses that buffer the level of this key metabolic intermediate in the light, for example, by decreasing starch synthesis, and by decreasing consumption of hexose phosphates for sucrose synthesis in the cytosol by allosteric and post-transitional inhibition of sucrose-phosphate synthase (Huber and Huber 1996; Smith and Stitt 2007; Lunn 2016).

A third finding was that an endogenous circadian rhythm may contribute to the diel changes in SnRK1 activity, at least in the middle of the 24-h cycle, when SnRK1 activity decreased between about ZT12 and ZT16. The decrease after ZT12 was partly retained in low continuous light (Fig. 3) and was also observed in plants growing in a T28 cycle, even though they were in the light between ZT12 and ZT14 (Fig. 4). The parallel changes in sugars and predicted extra-nuclear SnRK1 activity after returning plants from low to growth irradiance may also point to an endogenous signal that relays information about C status in the previous 24-h cycle.

### SnRK1 activity may be regulated by multiple metabolic inputs including Tre6P and hexose phosphates

The unexpected diel response pointed to SnRK1 operating within a network that controls C utilization in the daytime and at night to maintain C homeostasis. SnRK1 activity integrates various inputs including multiple signals from metabolism (Polge and Thomas 2007; Sheen and Baena-Gonzalez 2008; Wurzinger et al. 2018). We asked to what extent diel changes in SnRK1 activity are accompanied by reciprocal changes in the levels of sugars that might be viewed as indicators of C availability, or reciprocal changes in the levels of metabolites, like Tre6P, Glc6P, and Glc1P, that are known to inhibit SnRK1 in vitro (Toroser et al. 2000; Zhang et al. 2009; Debast et al. 2011; Delatte et al. 2011; Piattoni et al. 2011; Nunes et al. 2013b; Coello and Martínez-Barajas 2014; Emanuelle et al. 2015; Griffiths et al. 2016; Bledsoe et al. 2017; Baena-González and Lunn 2020; Henry et al. 2020).

The role of Tre6P in regulation of SnRK1 has attracted wide attention (Zhang et al. 2009, 2015; Nunes et al. 2013a; Henry et al. 2015; Bledsoe et al. 2017; Baena-González and Lunn 2020). To directly test whether an induced increase in Tre6P leads to inhibition of in vivo SnRK1 activity, we crossed our reporter constructs into lines carrying an inducible bacterial TPS construct. An induced 1.4- to 2-fold increase in Tre6P in the light period indeed led to 12%–23% decrease in phosphorylation of the NUC reporter polypeptide (Fig. 5), and a weak decrease for the GEN reporter polypeptide (Supplemental Fig. S6A). On the other hand, during diel cycles in wild-type plants Tre6P levels did not show a consistent negative relationship to NUC or GEN phosphorylation (Fig. 1; Table 1). Whereas Tre6P levels rose 3- to 5-fold during the light period, peaked at ZT12 (ED) and then fell, (Fig. 1B), SnRK1 activity did not change during the light period and, after darkening, fell by 2- to 3-fold to a minimum at around ZT16 (Fig. 1A). Also, comparison of the magnitude of the changes in Figs. 5 and 1 indicates that the diel changes of Tre6P are too small to explain the diel changes in NUC phosphorylation. In the last part of the night, where there was a negative correlation between Tre6P levels and SnRK1 activity (Supplemental Table S1), an induced increase in Tre6P unexpectedly resulted in an increase rather than a decrease in SnRK1 activity (Fig. 6). Whilst these findings provide evidence that Tre6P can contribute to the regulation of SnRK1 in vivo, they also show that the contribution of Tre6P is context dependent and that Tre6P is not the only or even the major factor that regulates SnRK1 activity.

The possible role of hexose phosphates like Glc6P and Glc1P in the in vivo regulation of SnRK1 activity has attracted less attention, despite evidence from in vitro assays that they can inhibit SnRK1 (Toroser et al. 2000; Zhang et al. 2009; Piattoni et al. 2011; Nunes et al. 2013b). Several features of our study point to them playing a role in vivo. First, visual inspection of diel changes under control conditions reveal that Glc6P and Glc1P often change in a reciprocal manner to SnRK1 activity (Fig. 1), peaking in the first part of the night at ZT16 at the same time as the minimum in SnRK1 phosphorylation activity. A negative relationship between SnRK1 and Glc6P was also observed when plants were exposed to a sudden low-light day (Fig. 2; see also Moraes et al. 2019) or transferred to continuous low light (Fig. 3; Supplemental Fig. S4). Second, there was a significant negative correlation between Glc6P or Glc1P levels and NUC or GEN phosphorylation in some, though not all, full diel cycles (Table 1) and highly consistent negative correlations in the night (Supplemental Table S1). This conclusion was supported by comparison of the rates of change of Glc6P or Glc1P levels and SnRK1 activity at different times in the diel cycle (Supplemental Table S3). Glc6P and Glc1P are linked by the near-equilibrium reaction catalyzed by phosphoglucomutase, with both changing in parallel and Glc6P being about 10-fold higher than Glc1P, therefore, the observed negative relationship between SnRK1 and Glc1P might be secondary to the relationship with Glc6P.

The question also arises what endogenous factor might be responsible for the decline in SnRK1 activity between ZT12 and ZT16, which occurred even when plants were not darkened at ZT12 (see above). One possible explanation is that this decline is driven by diel changes in starch turnover. As shown in Fernandez et al. (2017) and Ishihara et al. (2022), starch mobilization occurs simultaneously with starch synthesis in the light and speeds up with time in the light, leading from about ZT10 onwards to a marked rise in sucrose and related metabolites, including Tre6P. This might contribute to the decline in SnRK1 activity after ZT12 in continuous light. As suggested in Fernandez et al. (2017) and Ishihara et al. (2022), this gradual rise in the rate of starch mobilization is itself under circadian control. However, it would be premature to exclude the possibility that the circadian clock exerts a more direct influence on SnRK1 activity, mirroring the recent discovery that SnRK1 is involved in transcriptional regulation of the circadian clock (Jeong et al. 2015; Shin et al. 2017; Frank et al. 2018; Sánchez-villarreal et al. 2018). A possible link between starch mobilization and SnRK1 activity is also of interest in view of the report that the dark-expressed SnRK1β3 subunit binds maltose, pointing to a possible role in regulating starch degradation or of starch degradation, via maltose, regulating SnRK1 activity (Ruiz-Gayosso et al. 2018).

Taken together, our results point to both Tre6P and hexose phosphates contributing to the regulation of SnRK1 in vivo. These metabolites provide complementary information about C status. Tre6P provides information about the amount of sucrose (Lunn et al. 2006; Figueroa and Lunn 2016; Figueroa et al. 2016; Fichtner et al. 2017; Fichtner and Lunn 2021), which is a key long-distance transport metabolite and often a C-storage molecule (Lunn 2016). Hexose phosphate levels reflect the balance between several central metabolic pathways, for example, between photosynthetic C fixation and sucrose and starch synthesis in the light, and between starch mobilization, respiration, and biosynthesis in the dark. Further, Tre6P and hexose phosphates may have differing subcellular and intercellular compartmentation. TPS1 protein, and by implication Tre6P, is partly localized to the nucleus (Fichtner et al. 2020) whereas hexose phosphates are likely to have a more general subcellular distribution with high levels in the cytosol (Stitt et al. 1980; Gerhardt et al. 1987). Such differences in metabolite compartmentation might underlie some of the differing responses of the NUC and GEN reporters. Further, TPS1, and by implication Tre6P, is very abundant in companion cells and surrounding phloem parenchyma (Fichtner et al. 2020) whereas hexose phosphates are probably distributed more generally across cell types, including high levels in photosynthetic mesophyll cells. This may introduce important nuances into regulation of SnRK1 activity, whose detection would require cell-specific expression of the reporter constructs.

### Changes in complex composition might impact on diel SnRK1 activity

An additional regulatory mechanism that might contribute to observed diel changes in SnRK1 activity would be changes in subunit composition, including expression or stability of its subunits, such as by SUMOylation and ubiquitination of SnRK1α1, SnRK1β1, SnRK1β2 SnRK1β3, and by myristoylation of SnRK1β1 and SnRK1β2 (Ruiz-Gayosso et al. 2018; Blanco et al. 2019; Ramon et al. 2019; Wang et al. 2020). These were shown to affect cellular localization, primarily by translocating SnRK1 in and out of the nucleus. We cannot yet be certain whether changes in SnRK1 composition contribute to the diel changes in SnRK1 activity detected using the ACC reporter constructs. In the future, it would be of interest to compare changes in SnRK1 composition and SnRK1 location with the relative rates of NUC and GEN phosphorylation. Furthermore, the recent study of Van Leene et al. (2022) highlights that TPS Class II proteins can interact with and may inhibit SnRK1 activity. The authors also proposed that binding of TPS Class II proteins might favor redistribution out of the nucleus and toward the endoplasmic reticulum. To assess the contribution of this recently described mechanism to the diel regulation of SnRK1 activity will require more information about whether there are diel changes in TPS Class II protein abundance, and about whether Tre6P and other metabolites affect the interaction between TPS Class II proteins and SnRK1.

### Comparison of in vivo SnRK1 activity with responses of SnRK1 downstream targets highlights potential flexibility in downstream signaling pathways

As already mentioned, downstream target genes identified in protoplast overexpression studies have been widely used to make inferences about SnRK1 activity in vivo. We inspected published data to learn if the diel changes in transcript abundance of these genes are consistent with the diel changes of in vivo SnRK1 activity, as monitored by reporter peptide phosphorylation. To do this, we focused on studies in which Arabidopsis was grown in the same conditions as those used in the current study. As shown in Supplemental Fig. S9A, which replots data from Usadel et al. (2008), there was a fairly consistent pattern with the abundance of SnRK1-induced transcripts falling in the light period and rising during the night, and the abundance of SnRK1-repressed transcripts rising in the light and falling during the night (two exceptions were *AXP* and *EXP10*). This pattern contrasts with the response of the NUC or GEN reporter polypeptide phosphorylation, which was relatively high in the light period and fell in the first part of the night (Fig. 1A). In a second comparison, we inspected the response of a set of SnRK1 target transcripts after a sudden decrease in irradiance (Moraes et al. 2019), similar to the treatment in Fig. 2 and the first 12 h after transfer to continuous low light in Fig. 3 (Supplemental Fig. S9B). Transfer from 160 to 60 µmol m^−2^ s^−1^ led, for SnRK1-induced genes, to a small but consistent increase in *DIN6* and *BCAT2* and a large increase in *DIN1* transcript abundance and, for SnRK1-repressed genes, to a small but consistent decrease in *CPN60α* and *Asp.P* transcript abundance. This contrasts with NUC or GEN reporter polypeptide phosphorylation, which did not change after transfer to low irradiance (Figs. 2 and 3). In a recent study, Peixoto et al. (2021) reported that abundance of SnRK1-induced transcripts was maximal at the end of the night when Tre6P levels were lowest, and minimal at the end of the day when Tre6P levels peaked. However, even though the changes of Tre6P levels in Peixoto et al. (2021) resembled those in the current study, the responses of transcript abundance did not match the changes in NUC or GEN reporter polypeptide phosphorylation reported in the current study. Furthermore, whilst transient elevation of Tre6P in an inducible bacterial TPS line led to decreased abundance of transcripts for three SnRK1-induced genes (*BGAL*, *DIN10*, *PYL5*; Peixoto et al. 2021), consistent with involvement of Tre6P in diel regulation of SnRK1 signaling, it did not decrease transcript abundance for many other SnRK1-induced genes, such as *DIN1*, *DIN6*, *AXP*, and *EXP10*.

There are various possible explanations for discrepancies between SnRK1 activity as monitored by phosphorylation of the NUC or GEN reporter polypeptides and SnRK1 activity as inferred from the abundance of downstream transcripts. It is possible that in some cases, gene expression is driven by a fraction of the total SnRK1 pool, and that this fraction may remain undetectable when assaying total SnRK1 activities (Baena-González and Lunn 2020). The discrepancy may also reflect multiple levels of regulation, for example, not only via changes in SnRK1 activity itself but also via inputs that modify the affinity of SnRK1 for different immediate downstream targets or via inputs that potentiate or attenuate different downstream signaling pathways. Such modifying factors might also explain the varying responses of different SnRK1 downstream transcripts within a given experiment (see above, also Griffiths et al. 2016), as well as between different studies. Indeed, of the top SnRK1 target genes, only a few changed in a reproducible manner following SnRK1 induction in protoplasts versus whole plants (Baena-González et al. 2007; Ramon et al. 2019; Peixoto et al. 2021). For example, *DIN1*, *DIN6*, and *CAT1*, whose expression is commonly used as a readout of SnRK1 activity, are strongly upregulated in response to transient expression of SnRK1α1 in protoplasts (Baena-González et al. 2007), but remain unaffected in response to constitutive overexpression of SnRK1α1 in whole plants (Peixoto et al. 2021). Potential modifying factors might include TPS Class II proteins or, indeed, any of the numerous other proteins that interact with SnRK1 (Van Leene et al. 2022). It is also possible that, as has been shown to be the case in TOR signaling in mammals (Kang et al. 2013), the intrinsic capacity of a given phosphorylation site to serve as a substrate may differ from target to target, allowing flexible responses of different output pathways to a change in the basal activity or the composition of the SnRK1 complex.

Functionally, operation of SnRK1 in a benign diel cycle as well as during starvation mirrors the differing responses of two subsets of C-regulated genes. Many of these show large changes in expression during a regular equinoctial diel cycle (Smith and Stitt 2007; Usadel et al. 2008; Cookson et al. 2016; Flis et al. 2016). These genes may contribute to modifications of metabolism and slowing down of growth, and serve to maintain C homeostasis and avoid transient periods of C starvation and the resulting wasteful alternation between growth and catabolism (Ishihara et al. 2015b, 2017). Other C-regulated genes do not show changes in expression until starch is exhausted, and may serve to orchestrate catabolic responses in conditions when the plant can no longer avoid C starvation.

In conclusion, by employing an in vivo reporter polypeptide assay we have uncovered unexpected changes in SnRK1 activity under benign light-dark cycles. Continued SnRK1 activity in the light period may contribute to maintenance of diel sugar homeostasis, by restricting C utilization for growth and supporting build-up of C reserves. Further, whilst demonstrating that Tre6P can inhibit SnRK1 activity in vivo, we also show that the contribution of Tre6P is context dependent and that hexose phosphates and probably also other factors, including interacting proteins (Van Leene et al. 2022), interact to regulate SnRK1 activity and signaling.

## Materials and methods

### Plant material

All Arabidopsis (*A. thaliana* [L.] Heynh.) genotypes were in the Columbia-0 (Col-0) background. Lines expressing the NUC or GEN SnRK1-reporters were generated as described in Sanagi et al. (2021). The reporters comprise a 57-amino acid peptide derived from rat (*Rattus norvegicus*) acetyl CoA carboxylase (rACC1), containing the AMPK recognition motif and Ser79 phosphorylation site, fused at the C-terminus to eGFP and a double hemagglutinin tag (Supplemental Fig. S1; Deroover et al. 2016). The NUC reporter includes an N-terminal SV40 nuclear localization signal that targets the reporter specifically to the nucleus, while the GEN reporter has no added nuclear localization signal and is localized in both the nucleus and cytoplasm (Supplemental Fig. S2). The Arabidopsis (Col-0) ethanol-inducible TPS overexpressor (iTPS 29.2) and empty-vector control AlcR lines were the same as those described in Martins et al. (2013). The Arabidopsis (Col-0) 35S:SnRK1α1 overexpression line (Jossier et al. 2009) and the 35S:SnRK1α1^K48M^ line, expressing the SnRK1α1 subunit with a mutation that disrupts ATP binding (Cho et al. 2016), were kindly provided by Elena Baena-González (University of Oxford, UK) and Hsing-Yi Cho (National Chung-Hsing University, Academia Sinica, Taiwan), respectively.

### Introgression of NUC and GEN SnRK1 reporters

The NUC and GEN SnRK1 reporters were introgressed into established lines with altered SnRK1 activity or inducible expression of TPS by crossing. The respective homozygous parental lines were grown in soil for 4–5 wk (see below). Pollen from the parental parent was transferred by rubbing anthers from mature flowers against exposed stigmas of emasculated maternal plants under a binocular microscope, and the inflorescence meristems and unopened flower buds were removed. After crossing, the maternal plants were grown on, with regular watering, for 2–3 wk until siliques had fully developed and started to dry. Siliques were collected, left to dry for a further 3–4 d at room temperature and then kept at 10 °C until seeds were germinated on selective media. After surface sterilization by treatment with 2% (w/v) sodium hypochlorite for 5 min at room temperature and washing in five changes of water, seeds were placed on 0.5 × Murashige and Skoog medium (Murashige and Skoog 1962) containing 1% (w/v) sucrose and appropriate antibiotics for selection of the respective transgenes from each parent: NUC/GEN phosphinothricin (Basta); 35S:SnRK1α1, kanamycin; 35S: SnRK1α1^K48M^, hygromycin; iTPS/alcR; kanamycin. The seeds were stratified for 2 d at 4 °C in the dark and then transferred to 22 °C in continuous light at 100 µmol m^−2^ s^−1^. After 7 d, surviving seedlings were transferred to soil. The F1 plants were selfed and F2 progeny were screened on selective media, as above, to identify lines that exhibit the expected susceptible: resistant ratio of 7:9. Individual F2 plants were selfed and the F3 progeny were screened on selective media, as above, to identify lines that were homozygous for both transgenic loci.

### Plant growth and harvest

Seeds were sown on a 1:1 mixture of compost (Stender AG, Schermbeck, Germany; https://www.stender.de) and vermiculite in 6-cm diameter pots, covered and kept at 4 °C in the dark for 48 h then transferred to a controlled environment chamber (Percival E-36 L chamber, CLF Plant Climatics GmbH, Weringen, Germany; https://www.plantclimatics.de/) with a 16-h photoperiod (160 µmol m^−2^ s^−1^ irradiance provided by white LEDs) and day/night temperatures of 21 °C/19 °C. After germination, seedlings were transferred to 10-cm diameter pots (four or five seedlings per pot), and grown as above or as described for individual experiments. Whole rosettes were harvested in situ and immediately quenched in liquid nitrogen, with four or five plants from the same pot being pooled for each biological replicate. Frozen plant material was ground to a fine powder and stored at −80 °C until use.

### Metabolite extraction and analysis

Soluble sugars and starch were extracted and assayed enzymatically as described in Gibon et al. (2002) with three to four biological replicates per sample type. For each biological replicate, values were calculated as the average of two measurements. Tre6P, other phosphorylated intermediates and organic acids were extracted with chloroform-methanol and quantified by anion-exchange high-performance liquid chromatography coupled to tandem mass spectrometry as described in Lunn et al. (2006), with modifications as described in Figueroa et al. (2016). An exception was in the T28 cycle experiment (Supplemental Fig. S5 and Data Set S1, Experiment 5) for which the Glc6P content was measured enzymatically from the same ethanolic extracts used for sugar measurements.

### Ethanol-inducible iTPS experiments

After introgression of the NUC or GEN SnRK1 reporters, ethanol-inducible TPS (iTPS) and control alcR lines (Martins et al. 2013) were grown as described above. On the day of harvest, plants were sprayed with either water (mock-induction control) or with 2% (v/v) ethanol to induce expression of the bacterial TPS and thereby increase Tre6P levels. Samples were collected and harvested as described above.

### SDS-PAGE and immunoblot assay of SnRK1-reporter phosphorylation

Proteins were extracted from aliquots (10 mg FW) of frozen, finely powdered plant material by suspension in 100 µL of pre-warmed (90 °C) sample buffer (66 mM Tris-HCl, pH 6.8, 10% [v/v] glycerol, 2% [w/v] sodium dodecyl sulfate, 0.13% [w/v] bromophenol blue) quickly vortex mixed, incubated at 90 °C for 5 min, then chilled on ice and centrifuged at 13,500*×g* (4 °C) for 3 min. Aliquots of the supernatant containing approx. 12 µg of soluble protein were electrophoresed on a 12% (w/v) polyacrylamide-SDS gel. Proteins were electro-blotted onto nitrocellulose membrane, visualized by staining with 0.2% (w/v) Ponceau Red in 1% (v/v) acetic acid and imaged for use as a protein-loading reference. After blocking in Tris-buffered saline containing 5% (w/v) milk powder, the membrane was incubated overnight at 4 °C with rabbit anti-phospho-acetyl CoA carboxylase (Ser79) antibody (#3661; Cell Signaling Technology Europe BV, Leiden, The Netherlands; https://www.cellsignal.de) at a dilution of 1:1,500. After washing, NUC/GEN phosphorylation was detected by incubation with IRDye 800CW goat anti-rabbit IgG (H + L) secondary antibody (Li-COR Biosciences GmbH, Bad Homburg, Germany; www.licor.com) at a dilution of 1:9,000 and scanning with an Odyssey Imaging Scanner (Li-COR), and quantified using Image Studio Live 5.2 software (Li-COR). Total NUC/GEN polypeptide abundance was detected by probing duplicate blots with a mouse anti-GFP antibody (#11814460001; Sigma Aldrich (Roche), Darmstadt, Germany; www.sigmaaldrich.com) at a dilution of 1:1,500, followed by incubation with IRDye 680RD goat anti-mouse IgG (H + L) secondary antibody (Li-COR) at a dilution of 1:9,000, and quantified as above.

To minimize the impact of gel-to-gel variation, aliquots from an extended night sample were loaded onto each gel and the signal from this sample was used to normalize the signals from the other samples on the corresponding immunoblot. Two aliquots of the protein extract from each biological replicate were analyzed on separate gels and, after normalization as described above, the average of the two technical replicates was calculated as the reported value for that biological replicate. The signal ratio pACC/GFP was used to normalize for any differences in reporter protein abundance in the tissue and differences in gel loading. See Supplemental Fig. S1 for further details.

### Confocal microscopy

To determine the subcellular localization of the NUC and GEN reporter polypeptides, GFP was visualized by laser confocal microscopy of 6-d-old seedlings. Confocal imaging was performed on a Leica TCS SP5 microscope (Leica Microsystems, Wetzlar, Germany; https://www.leica-microsystems.com) using a 20× objective. To visualize nuclei, NUC samples were stained with 10 μg/mL propidium iodide (PI) solution for 5–10 min. A 488 nm laser (40% intensity) was used to excite GFP and fluorescence emission was detected in the 524–555 nm range with a gain setting of 860. A 561 nm laser (10% intensity) was used to excite PI and fluorescence emission was detected in the 571–640 nm range with a gain setting of 550. Confocal Z sections were acquired every 0.5 μm.

### Statistical analysis

Technical replicates were always averaged to generate a single value for each biological replicate, statistical analysis was restricted to biological replicates and performed using Sigma-Plot 14.5 software (Systat Software GmbH, Düsseldorf, Germany; http://www.systat.de). Significance of changes in metabolite and SnRK1 activity was tested by one-way ANOVA using a pairwise multiple comparison procedure, with post-hoc testing by the Holm–Sidak method (*P* < 0.05), as described in each figure legend.

## Supplementary Material

kiad066_Supplementary_Data

## Supplemental data

The following materials are available in the online version of this article.

**Supplemental Fig. S1**. In vivo SnRK1 activity assay

**Supplemental Fig. S2**. Localization of reporter peptides by confocal microscopy

**Supplemental Fig. S3**. Validation of the NUC and GEN constructs in whole Arabidopsis rosettes

**Supplemental Fig. S4**. Glc6P levels after a shift at dawn to continuous low light (supplementary to Fig. 3).

**Supplemental Fig. S5**. T28 and T24 cycles; diel Glc6P levels and comparison of SnRK1 activity after excluding time points affected by C starvation (supplementary to Fig. 4).

**Supplemental Fig. S6**. Response of NUC and GEN phosphorylation in crosses with a line containing inducible bacterial TPS (supplementary to Fig. 5).

**Supplemental Fig. S7**. Impact of transiently elevated Tre6P in the light period on the levels of selected metabolites (supplementary to Fig. 5).

**Supplemental Fig. S8**. Transient elevation of Tre6P at the end of the night slows down starch mobilization and leads to low glucose and high organic acid levels (supplementary to Fig. 6).

**Supplemental Fig. S9**. Transcript abundance for SnRK1 target genes during a diel cycle, after an extension of the night and after a sudden decrease in light intensity for 1 day.

**Supplemental Table S1**. Correlations between metabolite levels and NUC or GEN phosphorylation in unperturbed diel cycles, performed separately for the light period and the night (supplementary to Table 1 and Fig. 1).

**Supplemental Table S2**. Correlations between metabolite levels (supplementary to Table 1 and Fig. 1).

**Supplemental Table S3**. Correlations between changes in metabolite levels and changes in NUC or GEN phosphorylation (supplementary to Table 1 and Fig. 1).

**Supplemental Table S4**. Correlations between metabolite levels and NUC or GEN phosphorylation in growth regimes that differed from the equinoctial T24 cycle (supplementary to Figs. 3 and 4).

**Supplemental Table S5**. Summary of correlation analysis between changes in SnR, K1 NUC or GEN activity to changes in metabolite levels (supplementary to Figs. 3 and 4).

**Supplemental Table S6**. Correlation analysis between metabolite levels and NUC phosphorylation following transient elevation of Tre6P in the light period (supplementary to Fig. 5, Supplemental Data Set S2, Experiment 7).

**Supplemental Text S1.** Correlations between in vivo SnRK1 activity and metabolites in continuous light, in T28 cycles and in experiments where TPS is induced (supplementary to Results section “Correlation analysis of relationships between SnRK1 activity and metabolite levels’ and to Supplemental Tables S4–S6).

**Supplemental Data Set S1**. In vivo SnRK1 activity and metabolite levels.

**Supplemental Data Set S2**. Regressions between in vivo SnRK1 activity and metabolite levels.

## References

[kiad066-B1] Ávila-Castañeda A , Gutiérrez-Granados N, Ruiz-Gayosso A, Sosa-Peinado A, Martínez-Barajas E, Coello P. Structural and functional basis for starch binding in the SnRK1 subunits AKINβ2 and AKINβγ. Front Plant Sci. 2014:5:199 24904601 10.3389/fpls.2014.00199 PMC4032982

[kiad066-B2] Baena-González E , Lunn JE. SnRK1 and trehalose 6-phosphate – two ancient pathways converge to regulate plant metabolism and growth. Curr Opin Plant Biol. 2020:55:52–59 32259743 10.1016/j.pbi.2020.01.010

[kiad066-B3] Baena-González E , Rolland F, Thevelein JM, Sheen J. A central integrator of transcription networks in plant stress and energy signalling. Nature. 2007:448(7156):938–942 17671505 10.1038/nature06069

[kiad066-B4] Belda-Palazón B , Costa M, Beeckman T, Rolland F, Baena-González E. ABA represses TOR and root meristem activity through nuclear exit of the SnRK1 kinase. Proc Natl Acad Sci U S A. 2022:119(28):2204862119 10.1073/pnas.2204862119 PMC9282376 35787039

[kiad066-B5] Blanco NE , Liebsch D, Díaz MG, Strand Å, Whelan J. Dual and dynamic intracellular localization of *Arabidopsis thaliana* SnRK1.1. J Exp Bot. 2019:70(8):2325–2338 30753728 10.1093/jxb/erz023

[kiad066-B6] Bledsoe SW , Henry C, Griffiths CA, Paul MJ, Feil R, Lunn JE, Stitt M, Lagrimini LM. The role of Tre6P and SnRK1 in maize early kernel development and events leading to stress-induced kernel abortion. BMC Plant Biol. 2017:17(1):1–17 28403831 10.1186/s12870-017-1018-2 PMC5389189

[kiad066-B7] Borghi GL , Moraes TA, Günther M, Feil R, Mengin V, Lunn JE, Stitt M, Arrivault S. Relationship between irradiance and levels of Calvin–Benson cycle and other intermediates in the model eudicot Arabidopsis and the model monocot rice. J Exp Bot. 2019:70(20):5809–5825 31353406 10.1093/jxb/erz346 PMC6812724

[kiad066-B8] Broeckx T , Hulsmans S, Rolland F. The plant energy sensor: evolutionary conservation and divergence of SnRK1 structure, regulation, and function. J Exp Bot. 2016:67(22):6215–6252 27856705 10.1093/jxb/erw416

[kiad066-B9] Cabib E , Leloir LF. The biosynthesis of trehalose phosphate. J Biol Chem. 1958:231(1):259–275 13538966

[kiad066-B10] Cho H , Wen T, Wang Y, Shih M. Quantitative phosphoproteomics of protein kinase SnRK1 regulated protein phosphorylation in Arabidopsis under submergence. J Exp Bot. 2016:67(9):2745–2760 27029354 10.1093/jxb/erw107 PMC4861021

[kiad066-B11] Coello P , Martínez-Barajas E. The activity of SnRK1 is increased in *Phaseolus vulgaris* seeds in response to a reduced nutrient supply. Front Plant Sci. 2014:5:1–7 10.3389/fpls.2014.00196 PMC4030202 24860586

[kiad066-B12] Cookson S , Yadav UP, Klie S, Morcuende RM, Usadel B, Lunn JE, Stitt M. Temporal kinetics of the transcriptional response to carbon depletion and sucrose readdition in *Arabidopsis* seedlings. Plant Cell Environ. 2016:39(4):768–786 26386165 10.1111/pce.12642

[kiad066-B13] Crepin N , Rolland F. SnRK1 activation, signaling, and networking for energy homeostasis. Curr Opin Plant Biol. 2019:51:29–36 31030062 10.1016/j.pbi.2019.03.006

[kiad066-B14] Crozet P , Margalha L, Confraria A, Rodrigues A, Martinho C, Adamo M, Elias CA, Baena-González E. Mechanisms of regulation of SNF1/AMPK/SnRK1 protein kinases. Front Plant Sci. 2014:5:190 24904600 10.3389/fpls.2014.00190 PMC4033248

[kiad066-B15] Dale S , Wilson WA, Edelman AM, Hardie DG. Similar substrate recognition motifs for mammalian AMP-activated protein kinase, higher plant HMG-CoA reductase kinase-A, yeast SNF1, and mammalian calmodulin-dependent protein kinase I. FEBS Lett. 1995:361(2–3):191–195 7698321 10.1016/0014-5793(95)00172-6

[kiad066-B16] Debast S , Nunes-Nesi A, Hajirezaei MR, Hofmann J, Sonnewald U, Fernie AR, Börnke F. Altering trehalose-6-phosphate content in transgenic potato tubers affects tuber growth and alters responsiveness to hormones during sprouting. Plant Physiol. 2011:156(4):1754–1771 21670224 10.1104/pp.111.179903 PMC3149945

[kiad066-B17] Delatte TL , Sedijani P, Kondou Y, Matsui M, de Jong GJ, Somsen GW, Wiese-Klinkenberg A, Primavesi LF, Paul MJ, Schluepmann H. Growth arrest by trehalose-6-phosphate: an astonishing case of primary metabolite control over growth by way of the SnRK1 signaling pathway. Plant Physiol. 2011:157(1):160–174 21753116 10.1104/pp.111.180422 PMC3165867

[kiad066-B18] Delorge I , Figueroa CM, Feil R, Lunn JE, van Dijck P. Trehalose-6-phosphate synthase 1 is not the only active TPS in *Arabidopsis thaliana*. Biochem J. 2015:466(2):283–290 25495218 10.1042/BJ20141322

[kiad066-B19] Deroover S , Ghillebert R, Broeckx T, Winderickx J, Rolland F. Trehalose-6-phosphate synthesis controls yeast gluconeogenesis downstream and independent of SNF1. FEMS Yeast Res. 2016:16(4):fow036 10.1093/femsyr/fow036 27189362

[kiad066-B20] Emanuelle S , Hossain MI, Moller IE, Pedersen HL, van de Meene AM, Doblin MS, Koay A, Oakhill JS, Scott JW, Willats WG, et al SnRK1 from *Arabidopsis thaliana* is an atypical AMPK. Plant J. 2015:82(2):183–192 25736509 10.1111/tpj.12813

[kiad066-B21] Fernandez O , Ishihara H, George GM, Mengin V, Flis A, Sumner D, Arrivault S, Feil R, Lunn JE, Zeeman SC, et al Leaf starch turnover occurs in long days and in falling light at the end of the day. Plant Physiol. 2017:174(4):2199–2212 28663333 10.1104/pp.17.00601 PMC5543966

[kiad066-B22] Fichtner F , Barbier FF, Feil R, Watanabe M, Annunziata MG, Stitt M, Beveridge CA, Lunn JE. Trehalose 6-phosphate is involved in triggering axillary bud outgrowth in garden pea (*Pisum sativum* L.). Plant J. 2017:92(4):611–623 28869799 10.1111/tpj.13705

[kiad066-B23] Fichtner F , Lunn JE. The role of trehalose 6-phosphate (Tre6P) in plant metabolism and development. Annu Rev Plant Biol. 2021:72(1):737–760 33428475 10.1146/annurev-arplant-050718-095929

[kiad066-B24] Fichtner F , Olas JJ, Feil R, Watanabe M, Krause U, Hoefgen R, Stitt M, Lunn JE. Functional features of TREHALOSE-6-PHOSPHATE SYNTHASE1, an essential enzyme in Arabidopsis. Plant Cell. 2020:32(6):1949–1972 32276986 10.1105/tpc.19.00837 PMC7268806

[kiad066-B25] Figueroa CM , Feil R, Ishihara H, Watanabe M, Kölling K, Krause U, Höhne M, Encke B, Plaxton WC, Zeeman SC, et al Trehalose 6-phosphate coordinates organic and amino acid metabolism with carbon availability. Plant J. 2016:85(3):410–423 26714615 10.1111/tpj.13114

[kiad066-B26] Figueroa CM , Lunn JE. A tale of two sugars: trehalose 6-phosphate and sucrose. Plant Physiol. 2016:172(1):7–27 27482078 10.1104/pp.16.00417 PMC5074632

[kiad066-B27] Flis A , Mengin V, Ivakov AA, Mugford ST, Hubberten HM, Encke B, Krohn N, Höhne M, Feil R, Hoefgen R, et al Multiple circadian clock outputs regulate diel turnover of carbon and nitrogen reserves. Plant Cell Environ. 2019:42(2):549–573 30184255 10.1111/pce.13440

[kiad066-B28] Flis A , Sulpice R, Abel C, Ivakov A, Liput M, Millar AJ, Stitt M. Photoperiod-dependent changes in the phase of core clock transcripts, and in global transcriptional outputs at dusk and dawn in *Arabidopsis*. Plant Cell Environ. 2016:39(9):1955–1981 27075884 10.1111/pce.12754

[kiad066-B29] Frank A , Matiolli CC, Viana AJC, Hearn TJ, Kusakina J, Belbin FE, Wells Newman D, Yochikawa A, Cano-Ramirez DL, Chembath A, et al Circadian entrainment in Arabidopsis by the sugar-responsive transcription factor bZIP63. Curr Biol. 2018:28(16):2597–2606.e6 30078562 10.1016/j.cub.2018.05.092 PMC6108399

[kiad066-B30] García-Salcedo R , Lubitz T, Beltran G, Elbing K, Tian Y, Frey S, Wolkenhauer O, Krantz M, Klipp E, Hohmann S. Glucose de-repression by yeast AMP-activated protein kinase SNF1 is controlled via at least two independent steps. FEBS J. 2014:281(7):1901–1917 24529170 10.1111/febs.12753

[kiad066-B31] Gerhardt R , Stitt M, Heldt HW. Subcellular metabolite levels in spinach leaves: regulation of sucrose synthesis during diurnal alterations in photosynthetic partitioning. Plant Physiol. 1987:83(2):399–407 16665257 10.1104/pp.83.2.399 PMC1056369

[kiad066-B32] Gibon Y , Bläsing OE, Palacios-Rojas N, Pankovic D, Hendriks JHM, Fisahn J, Höhne M, Günther M, Stitt M. Adjustment of diurnal starch turnover to short days: depletion of sugar during the night leads to a temporary inhibition of carbohydrate utilization, accumulation of sugars and post-translational activation of ADP-glucose pyrophosphorylase in the following light period. Plant J. 2004:39(6): 847–862 15341628 10.1111/j.1365-313X.2004.02173.x

[kiad066-B33] Gibon Y , Vigeolas H, Tiessen A, Geigenberger P, Stitt M. Sensitive and high throughput metabolite assays for inorganic pyrophosphate, ADPGlc, nucleotide phosphates, and glycolytic intermediates based on a novel enzymic cycling system. Plant J. 2002:30(2):221–235 12000458 10.1046/j.1365-313x.2001.01278.x

[kiad066-B34] Glab N , Oury C, Guérinier T, Domenichini S, Crozet P, Thomas M, Vidal J, Hodges M. The impact of *Arabidopsis thaliana* SNF1-related-kinase 1 (SnRK1)-activating kinase 1 (SnAK1) and SnAK2 on SnRK1 phosphorylation status: characterization of a SnAK double mutant. Plant J. 2017:89(5):1031–1041 27943466 10.1111/tpj.13445

[kiad066-B35] Glinski M , Weckwerth W. Differential multisite phosphorylation of the trehalose-6-phosphate synthase gene family in *Arabidopsis thaliana*: a mass spectrometry-based process for multiparallel peptide library phosphorylation analysis. Mol Cell Proteomics. 2005:4(10):1614–1625 16030010 10.1074/mcp.M500134-MCP200

[kiad066-B36] Goddijn OJM , van Dun K. Trehalose metabolism in plants. Trends Plant Sci. 1999:4(8):315–319 10431221 10.1016/s1360-1385(99)01446-6

[kiad066-B37] Graf A , Schlereth A, Stitt M, Smith AM. Circadian control of carbohydrate availability for growth in *Arabidopsis* plants at night. Proc Natl Acad Sci U S A. 2010:107(20):9458–9463 20439704 10.1073/pnas.0914299107 PMC2889127

[kiad066-B38] Griffiths CA , Sagar R, Geng Y, Primavesi LF, Patel MK, Passarelli MK, Gilmore IS, Steven RT, Bunch J, Paul MJ, et al Chemical intervention in plant sugar signalling increases yield and resilience. Nature. 2016:540(7634):574–578 27974806 10.1038/nature20591

[kiad066-B39] Hara LEO , Paul MJ, Wingler A. How do sugars regulate plant growth and development? New insight into the role of trehalose-6-phosphate. Mol Plant. 2013:6(2):261–274 23100484 10.1093/mp/sss120

[kiad066-B40] Hardie DG , Carling D. The AMP-activated protein kinase – fuel gauge of the mammalian cell? Eur J Biochem. 1997:246(2):259–273 9208914 10.1111/j.1432-1033.1997.00259.x

[kiad066-B41] Hardie DG , Carling D, Carlson M. The AMP-activated/SNF1 protein kinase subfamily: metabolic sensors of the eukaryotic cell? Annu Rev Biochem. 1998:67(1):821–855 9759505 10.1146/annurev.biochem.67.1.821

[kiad066-B42] Harthill JE , Meek SEM, Morrice N, Peggie MW, Borch J, Wong BHC, Mackintosh C. Phosphorylation and 14-3-3 binding of Arabidopsis trehalose-phosphate synthase 5 in response to 2-deoxyglucose. Plant J. 2006:47(2):211–223 16771775 10.1111/j.1365-313X.2006.02780.x

[kiad066-B43] Henninger M , Pedrotti L, Krischke M, Draken J, Wildenhain T, Fekete A, Rolland F, Müller MJ, Fröschel C, Weiste C, et al The evolutionarily conserved kinase SnRK1 orchestrates resource mobilization during Arabidopsis seedling establishment. Plant Cell. 2022:34(1):616–632 34755865 10.1093/plcell/koab270 PMC8774017

[kiad066-B44] Henry C , Bledsoe SW, Griffiths CA, Kollman A, Paul MJ, Sakr S, Lagrimini LM. Differential role for trehalose metabolism in salt-stressed maize. Plant Physiol. 2015:169(2):1072–1089 26269545 10.1104/pp.15.00729 PMC4587459

[kiad066-B45] Henry C , Watson-Lazowski A, Oszvald M, Griffiths C, Paul MJ, Furbank RT, Ghannoum O. Sugar sensing responses to low and high light in leaves of the C_4_ model grass *Setaria viridis*. J Exp Bot. 2020:71(3): 1039–1052 31677263 10.1093/jxb/erz495

[kiad066-B46] Herzig S , Shaw RJ. AMPK: guardian of metabolism and mitochondrial homeostasis. Nat Rev Mol Cell Biol. 2018:19(2):121–135 28974774 10.1038/nrm.2017.95 PMC5780224

[kiad066-B47] Huber SC , Huber JL. Role and regulation of sucrose-phosphate synthase in higher plants. Annu Rev Plant Physiol Plant Mol Biol. 1996:47(1):431–444 15012296 10.1146/annurev.arplant.47.1.431

[kiad066-B48] Hulsmans S , Rodriguez M, de Coninck B, Rolland F. The SnRK1 energy sensor in plant biotic interactions. Trends Plant Sci. 2016:21(8):648–661 27156455 10.1016/j.tplants.2016.04.008

[kiad066-B49] Hwang G , Kim S, Cho J-Y, Paik I, Kim J-I, Oh E. Trehalose-6-phosphate signaling regulates thermoresponsive hypocotyl growth in *Arabidopsis thaliana*. EMBO Rep. 2019:20(10):47828 10.15252/embr.201947828 PMC6776909 31393060

[kiad066-B50] Ishihara H , Alseekh S, Feil R, Perera P, George GM, Niedźwiecki P, Arrivault S, Zeeman SC, Fernie AR, Lunn JE, et al Rising rates of starch degradation with time in the light and trehalose 6-phosphate levels optimize carbon availability and thereby buffer growth. Plant Physiol. 2022:189(4):1976–2000 35486376 10.1093/plphys/kiac162 PMC9342969

[kiad066-B51] Ishihara H , Moraes T, Pyl E-T, Schulze WX, Obata T, Scheffel A, Fernie AR, Sulpice R, Stitt M. Growth rate correlates negatively with protein turnover in Arabidopsis accessions. Plant J. 2017:91(3):416–429 28419597 10.1111/tpj.13576

[kiad066-B52] Ishihara H , Obata T, Sulpice R, Fernie AR, Stitt M. Quantitative determination of the rates of protein synthesis and turnover in Arabidopsis rosettes using dynamic ^13^CO_2_ labelling and analysis of isotope enrichment of individual amino acids in free pools and in protein. Plant Physiol. 2015a:168(1):74–93 25810096 10.1104/pp.15.00209 PMC4424029

[kiad066-B53] Ishihara H , Obata T, Sulpice R, Fernie AR, Stitt M. Quantifying protein synthesis and degradation in arabidopsis by dynamic ^13^CO_2_ labeling and analysis of enrichment in individual amino acids in their free pools and in protein. Plant Physiol. 2015b:168(1):74–93 25810096 10.1104/pp.15.00209 PMC4424029

[kiad066-B54] Ivakov A , Flis A, Apelt F, Fünfgeld M, Scherer U, Stitt M, Kragler F, Vissenberg K, Persson S, Suslov D. Cellulose synthesis and cell expansion are regulated by different mechanisms in growing arabidopsis hypocotyls. Plant Cell. 2017:29(6):1305–1315 28550150 10.1105/tpc.16.00782 PMC5502445

[kiad066-B55] Jeong EY , Seo PJ, Woo JC, Park CM. AKIN10 delays flowering by inactivating IDD8 transcription factor through protein phosphorylation in Arabidopsis. BMC Plant Biol. 2015:15(1):1–13 25929516 10.1186/s12870-015-0503-8 PMC4416337

[kiad066-B56] Jossier M , Bouly JP, Meimoun P, Arjmand A, Lessard P, Hawley S, Hardie GD, Thomas M. SnRK1 (SNF1-related kinase 1) has a central role in sugar and ABA signalling in *Arabidopsis thaliana*. Plant J. 2009:59(2):316–328 19302419 10.1111/j.1365-313X.2009.03871.x

[kiad066-B57] Kang SA , Pacold ME, Cervantes CL, Lim D, Lou HJ, Ottina A, Gray NS, Turk BE, Yaffe MB, Sabatii DM. mTORC1 phosphorylation sites encode their sensitivity to starvation and rapamycin. Science. 2013:341(6144):1236566 10.1126/science.1236566 PMC3771538 23888043

[kiad066-B58] Kretzschmar T , Pelayo MAF, Trijatmiko KR, Gabunada LF, Alam R, Jimenez R, Mendioro MS, Slamet-Loedin IH, Sreenivasulu N, Bailey-Serres J, et al A trehalose-6-phosphate phosphatase enhances anaerobic germination tolerance in rice. Nat Plants. 2015:1(9):15124 27250677 10.1038/nplants.2015.124

[kiad066-B59] Lastdrager J , Hanson J, Smeekens S. Sugar signals and the control of plant growth and development. J Exp Bot. 2014:65(3):799–807 24453229 10.1093/jxb/ert474

[kiad066-B60] Leyman B , van Dijck P, Thevelein JM. An unexpected plethora of trehalose biosynthesis genes in *Arabidopsis thaliana*. Trends Plant Sci. 2001:6(11):510–513 11701378 10.1016/s1360-1385(01)02125-2

[kiad066-B61] Locke JCW , Millar AJ, Turner MS. Modelling genetic networks with noisy and varied experimental data: the circadian clock in *Arabidopsis thaliana*. J Theor Biol. 2005:234(3):383–393 15784272 10.1016/j.jtbi.2004.11.038

[kiad066-B62] Lu C , Lin C, Lee K, Chen J, Huang L, Ho S, Liu H, Hsing Y, Yu S. The SnRK1A protein kinase plays a key role in sugar signaling during germination and seedling growth of rice. Plant Cell. 2007:19(8):2484–2499 17766403 10.1105/tpc.105.037887 PMC2002608

[kiad066-B63] Lunn JE . Gene families and evolution of trehalose metabolism in plants. Funct Plant Biol. 2007:34(6):550–563 32689383 10.1071/FP06315

[kiad066-B64] Lunn JE . Sucrose metabolismEncyclopedia of Life Sciences (ELS). Hoboken, NJ: John Wiley Sons; 2016. p. 1–9

[kiad066-B65] Lunn JE , Delorge I, Mar C, van Dijck P, Stitt M. Trehalose metabolism in plants. Plant J. 2014:4(4):544–567 10.1111/tpj.12509 24645920

[kiad066-B66] Lunn JE , Feil R, Hendriks JHM, Gibon Y, Morcuende R, Osuna D, Scheible WR, Carillo P, Hajirezaei MR, Stitt M. Sugar-induced increases in trehalose 6-phosphate are correlated with redox activation of ADPglucose pyrophosphorylase and higher rates of starch synthesis in *Arabidopsis thaliana*. Biochem J. 2006:397(1):139–148 16551270 10.1042/BJ20060083 PMC1479759

[kiad066-B67] Ma J , Hanssen M, Lundgren K, Hernández L, Delatte T, Ehlert A, Liu CM, Schluepmann H, Dröge-Laser W, Moritz T, et al The sucrose-regulated Arabidopsis transcription factor bZIP11 reprograms metabolism and regulates trehalose metabolism. New Phytol. 2011:191(3):733–745 21534971 10.1111/j.1469-8137.2011.03735.x

[kiad066-B68] Mair A , Pedrotti L, Wurzinger B, Anrather D, Simeunovic A, Weiste C, Valerio C, Dietrich K, Kirchler T, Nägele T, et al SnRK1-triggered switch of bZIP63 dimerization mediates the low-energy response in plants. Elife. 2015:4:05828 10.7554/eLife.05828 PMC4558565 26263501

[kiad066-B69] Margalha L , Confraria A. SnRK1 and TOR: modulating growth – defense trade-offs in plant stress responses. J Exp Bot. 2019:70(8):2261–2274 30793201 10.1093/jxb/erz066

[kiad066-B70] Martins MCM , Hejazi M, Fettke J, Steup M, Feil R, Krause U, Arrivault S, Vosloh D, Figueroa CM, Ivakov A, et al Feedback inhibition of starch degradation in arabidopsis leaves mediated by trehalose 6-phosphate. Plant Physiol. 2013:163(3):1142–1163 24043444 10.1104/pp.113.226787 PMC3813640

[kiad066-B71] Martínez-Barajas E , Delatte T, Schluepmann H, de Jong GJ, Somsen GW, Nunes C, Primavesi LF, Coello P, Mitchell RA, Paul MJ. Wheat grain development is characterized by remarkable trehalose 6-phosphate accumulation pregrain filling: tissue distribution and relationship to SNF1-related protein kinase1 activity. Plant Physiol. 2011:156(1):373–381 21402798 10.1104/pp.111.174524 PMC3091070

[kiad066-B72] Mayer FV , Heath R, Underwood E, Sanders MJ, Carmena D, McCartney RR, Leiper FC, Xiao B, Jing C, Walker PA, et al ADP regulates SNF1, the *Saccharomyces cerevisiae* homolog of AMP-activated protein kinase. Cell Metab. 2011:14(5):707–714 22019086 10.1016/j.cmet.2011.09.009 PMC3241989

[kiad066-B73] Mengin V , Pyl ET, Moraes TA, Sulpice R, Krohn N, Encke B, Stitt M. Photosynthate partitioning to starch in *Arabidopsis thaliana* is insensitive to light intensity but sensitive to photoperiod due to a restriction on growth in the light in short photoperiods. Plant Cell Environ. 2017:40(11):2608–2627 28628949 10.1111/pce.13000

[kiad066-B74] Millar AJ , Carré IA, Strayer CA, Chua NH, Kay SA. Circadian clock mutants in *Arabidopsis* identified by luciferase imaging. Science. 1995:267(5201):1161–1163 7855595 10.1126/science.7855595

[kiad066-B75] Moraes TA , Mengin V, Annunziata MG, Encke B, Krohn N, Höhne M, Stitt M. Response of the circadian clock and diel starch turnover to one day of low light or low CO_2_. Plant Physiol. 2019:179(4):1457–1478 30670603 10.1104/pp.18.01418 PMC6446786

[kiad066-B76] Muralidhara P , Weiste C, Collani S, Krischke M, Kreisz P, Draken J, Feil R, Mair A, Teige M, Müller MJ, et al Perturbations in plant energy homeostasis prime lateral root initiation via SnRK1-bZIP63-ARF19 signalling. Proc Natl Acad Sci U S A. 2021:118(37):210696111 10.1073/pnas.2106961118 PMC8449399 34504003

[kiad066-B77] Murashige T , Skoog F. A revised medium for rapid growth and bio assays with tobacco tissue cultures. Physiol Plant. 1962:15(3):473–497

[kiad066-B78] Nietzsche M , Landgraf R, Tohge T, Börnke F. A protein-protein interaction network linking the energy-sensor kinase SnRK1 to multiple signaling pathways in *Arabidopsis thaliana*. Curr Opin Plant Biol. 2016:5:36–44

[kiad066-B79] Nietzsche M , Schießl I, Börnke F, Griffiths CA. The complex becomes more complex: protein-protein interactions of SnRK1 with DUF581 family proteins provide a framework for cell- and stimulus type-specific SnRK1 signaling in plants. Front Plant Sci. 2014:5:1–13 10.3389/fpls.2014.00054 PMC3930858 24600465

[kiad066-B80] Nuccio ML , Wu J, Mowers R, Zhou HP, Meghji M, Primavesi LF, Paul MJ, Chen X, Gao Y, Haque E, et al Expression of trehalose-6-phosphate phosphatase in maize ears improves yield in well-watered and drought conditions. Nat Biotech. 2015:33(8):862–869 10.1038/nbt.3277 26473199

[kiad066-B81] Nukarinen E , Nägele T, Pedrotti L, Wurzinger B, Baena-González E, Dröge-Laser W, Weckwerth W. Quantitative phosphoproteomics reveals the role of the AMPK plant ortholog SnRK1 as a metabolic master regulator under energy deprivation. Sci Rep. 2016:6(1):31697 27545962 10.1038/srep31697 PMC4992866

[kiad066-B82] Nunes C , O’Hara LE, Primavesi LF, Delatte TL, Schluepmann H, Somsen GW, Silva AB, Fevereiro PS, Wingler A, Paul MJ. The trehalose 6-phosphate/SnRK1 signaling pathway primes growth recovery following relief of sink limitation. Plant Physiol. 2013a:162(3):1720–1732 23735508 10.1104/pp.113.220657 PMC3707538

[kiad066-B83] Nunes C , Primavesi LF, Patel MK, Martinez-Barajas E, Powers SJ, Sagar R, Fevereiro PS, Davis BG, Paul MJ. Inhibition of SnRK1 by metabolites: tissue-dependent effects and cooperative inhibition by glucose 1-phosphate in combination with trehalose 6-phosphate. Plant Physiol Biochem. 2013b:63:89–98 23257075 10.1016/j.plaphy.2012.11.011

[kiad066-B84] Oszvald M , Primavesi LF, Grif A, Cohn J, Basu S, Nuccio ML, Paul MJ. Trehalose 6-phosphate regulates photosynthesis and assimilate partitioning in reproductive tissue. Plant Physiol. 2018:176(4):2623–2638 29437777 10.1104/pp.17.01673 PMC5884609

[kiad066-B85] Paul MJ , Gonzalez-Uriarte A, Grif CA, Hassani-Pak K. The role of trehalose 6-phosphate in crop yield and resilience. Plant Physiol. 2018:177(1):12–23 29592862 10.1104/pp.17.01634 PMC5933140

[kiad066-B86] Paul MJ , Watson A, Griffiths CA. Trehalose 6-phosphate signalling and impact on crop yield. Biochem Soc Trans. 2020:48(5):2127–2137 33005918 10.1042/BST20200286 PMC7609034

[kiad066-B87] Pedrotti L , Weiste C, Nägele T, Wolf E, Lorenzin F, Dietrich K, Mair A, Weckwerth W, Teige M, Baena-González E, et al Snf1-RELATED KINASE1-controlled C/S_1_-bZIP signaling activates alternative mitochondrial metabolic pathways to ensure plant survival in extended darkness. Plant Cell. 2018:30(2):495–509 29348240 10.1105/tpc.17.00414 PMC5868691

[kiad066-B88] Peixoto B , Moraes TA, Mengin V, Margalha L, Vicente R, Feil R, Höhne M, Sousa AGG, Lilue J, Stitt M, et al Impact of the SnRK1 protein kinase on sucrose homeostasis and the transcriptome during the diel cycle. Plant Physiol. 2021:187(3):1357–1373 34618060 10.1093/plphys/kiab350 PMC8566312

[kiad066-B89] Piattoni CV , Bustos DM, Guerrero SA, Iglesias AÁ. Nonphosphorylating glyceraldehyde-3-phosphate dehydrogenase is phosphorylated in wheat endosperm at serine-404 by an SNF1-related protein kinase. Plant Physiol. 2011:156(3):1337–1350 21546456 10.1104/pp.111.177261 PMC3135918

[kiad066-B90] Polge C , Jossier M, Crozet P, Gissot L, Thomas M. *β*-Subunits of the SnRK1 complexes share a common ancestral function together with expression and function specificities; physical interaction with nitrate reductase specifically occurs via AKIN*β*1-subunit. Plant Physiol. 2008:148(3):1570–1582 18768910 10.1104/pp.108.123026 PMC2577271

[kiad066-B91] Polge C , Thomas M. SNF1/AMPK/SnRK1 Kinases, global regulators at the heart of energy control? Trends Plant Sci. 2007:12(1):20–28 17166759 10.1016/j.tplants.2006.11.005

[kiad066-B92] Ponnu J , Wahl V, Schmid M. Trehalose-6-phosphate: connecting plant metabolism and development. Front Plant Sci. 2011:2:70 22639606 10.3389/fpls.2011.00070 PMC3355582

[kiad066-B93] Ramon M , Dang TVT, Broeckx T, Hulsmans S, Crepin N, Sheen J, Rolland F. Default activation and nuclear translocation of the plant cellular energy sensor SnRK1 regulate metabolic stress responses and development. Plant Cell. 2019:31(7):1614–1632 31123051 10.1105/tpc.18.00500 PMC6635846

[kiad066-B94] Randall RS . The plant AlcR-pAlcA ethanol-inducible system displays gross growth artefacts independently of downstream pAlcA-regulated inducible constructs. Sci Rep. 2021:11(1):1–5 33495493 10.1038/s41598-020-80903-z PMC7835360

[kiad066-B95] Rodrigues A , Adamo M, Crozet P, Margalha L, Confraria A, Martinho C, Elias A, Rabissi A, Lumbreras V, González-Guzmán M, et al ABI1 And PP2CA phosphatases are negative regulators of Snf1-related protein kinase1 signaling in *Arabidopsis*. Plant Cell. 2013:25(10):3871–3884 24179127 10.1105/tpc.113.114066 PMC3877788

[kiad066-B96] Ruiz-Gayosso A , Rodríguez-Sotres R, Martínez-Barajas E, Coello P. A role for the carbohydrate-binding module (CBM) in regulatory SnRK1 subunits: the effect of maltose on SnRK1 activity. Plant J. 2018:96(1):163–175 30003611 10.1111/tpj.14026

[kiad066-B97] Ryabova LA , Robaglia C, Meyer C. Target of rapamycin kinase: central regulatory hub for plant growth and metabolism. J Exp Bot. 2019:70(8):2211–2216 30984977 10.1093/jxb/erz108 PMC6463030

[kiad066-B98] Sanagi M , Aoyama S, Kubo A, Lu Y, Sato Y, Ito S, Abe M, Mitsuda N, Ohme-Takagi M, Kiba T, et al Low nitrogen conditions accelerate flowering by modulating the phosphorylation state of FLOWERING BHLH 4 in *Arabidopsis*. Proc Natl Acad Sci U S A. 2021:118(19):2022942118 10.1073/pnas.2022942118 PMC8126780 33963081

[kiad066-B99] Sánchez-Villarreal A , Davis AM, Davis SJ. AKIN10 activity as a cellular link between metabolism and circadian-clock entrainment in *Arabidopsis thaliana*. Plant Signal Behav. 2018:13(3):e1411448 10.1080/15592324.2017.1411448 PMC5927675 29231782

[kiad066-B100] Schluepmann H , Paul M. Trehalose metabolites in Arabidopsis—elusive, active and central. Arabidopsis Book. 2009:7:e0122 22303248 10.1199/tab.0122 PMC3243345

[kiad066-B101] Schluepmann H , Pellny T, van Dijken A, Smeekens S, Paul M. Trehalose 6-phosphate is indispensable for carbohydrate utilization and growth in *Arabidopsis thaliana*. Proc Natl Acad Sci U S A. 2003:100(11):6849–6854 12748379 10.1073/pnas.1132018100 PMC164535

[kiad066-B102] Sheen J , Baena-González E. Convergent energy and stress signaling. Trends Plant Sci. 2008:13(9):474–482 18701338 10.1016/j.tplants.2008.06.006 PMC3075853

[kiad066-B103] Shin J , Sánchez-Villarreal A, Davis AM, Du SX, Berendzen KW, Koncz C, Ding Z, Li C, Davis SJ. The metabolic sensor AKIN10 modulates the *Arabidopsis* circadian clock in a light-dependent manner. Plant Cell Environ. 2017:40(7):997–1008 28054361 10.1111/pce.12903

[kiad066-B104] Smith AM , Stitt M. Coordination of carbon supply and plant growth. Plant Cell Environ. 2007:30(9):1126–1149 17661751 10.1111/j.1365-3040.2007.01708.x

[kiad066-B105] Stitt M , Wirtz W, Heldt HW. Metabolite levels during induction in the chloroplast and extrachloroplast compartments of spinach protoplasts. Biochim Biophys Acta Bioenerg. 1980:593(1):85–102 10.1016/0005-2728(80)90010-9 7426648

[kiad066-B106] Sulpice R , Flis A, Ivakov AA, Apelt F, Krohn N, Encke B, Abel C, Feil R, Lunn JE, Stitt M, et al Arabidopsis coordinates the diurnal regulation of carbon allocation and growth across a wide range of photoperiods. Mol Plant. 2014:7(1):137–155 24121291 10.1093/mp/sst127

[kiad066-B107] Toroser D , Plaut Z, Huber SC. Regulation of a plant SNF1-related protein kinase by glucose-6-phosphate. Plant Physiol. 2000:123(1):403–412 10806257 10.1104/pp.123.1.403 PMC59014

[kiad066-B108] Usadel B , Bläsing OE, Gibon Y, Retzlaff K, Höhne M, Günther M, Stitt M. Global transcript levels respond to small changes of the carbon status during progressive exhaustion of carbohydrates in arabidopsis rosettes. Plant Physiol. 2008:146(4):1834–1861 18305208 10.1104/pp.107.115592 PMC2287354

[kiad066-B109] Vandesteene L , López-Galvis L, Vanneste K, Feil R, Maere S, Lammens W, Rolland F, Lunn JE, Avonce N, Beeckman T, et al Expansive evolution of the *TREHALOSE-6-PHOSPHATE PHOSPHATASE* gene family in Arabidopsis. Plant Physiol. 2012:160(2):884–896 22855938 10.1104/pp.112.201400 PMC3461562

[kiad066-B110] Vandesteene L , Ramon M, Le K, van Dijck P, Rolland F. A single active trehalose-6-P synthase (TPS) and a family of putative regulatory TPS-like proteins in Arabidopsis. Mol Plant. 2010:3(2):406–419 20100798 10.1093/mp/ssp114

[kiad066-B111] Van Leene Y , Eeckhout D, Gadeyne A, Matthijs C, Han C, De Winne N, Persiau G, Van De Slijke E, Persyn F, Mertens T, et al Mapping of the plant SnRK1 kinase signalling network reveals a key regulatory role for the class II T6P synthase-like proteins. Nat Plants. 2022:8(11):1245–1261 36376753 10.1038/s41477-022-01269-w

[kiad066-B112] Wahl V , Ponnu J, Schlereth A, Arrivault S, Langenecker T, Franke A, Feil R, Lunn JE, Stitt M, Schmid M. Regulation of flowering by trehalose-6-phosphate signaling in *Arabidopsis thaliana*. Science. 2013:339(6120):704–707 23393265 10.1126/science.1230406

[kiad066-B113] Wang Y , Wang L, Micallef BJ, Tetlow IJ, Mullen RT, Feil R, Lunn JE, Emes MJ. AKINβ1, a subunit of SnRK1, regulates organic acid metabolism and acts as a global modulator of genes involved in carbon, lipid, and nitrogen metabolism. J Exp Bot. 2020:71(3): 1010–1028 31624846 10.1093/jxb/erz460

[kiad066-B114] Wingler A , Delatte TL, Hara LEO, Primavesi LF, Jhurreea D, Paul MJ, Schluepmann H. Trehalose 6-phosphate is required for the onset of leaf senescence associated with high carbon availability. Plant Physiol. 2012:158(3):1241–1251 22247267 10.1104/pp.111.191908 PMC3291265

[kiad066-B115] Wurzinger B , Nukarinen E, Nägele T, Weckwerth W, Teige M. The SnRK1 kinase as central mediator of energy signaling between different organelles. Plant Physiol. 2018:176(2):1085–1094 29311271 10.1104/pp.17.01404 PMC5813556

[kiad066-B116] Yang HL , Liu YJ, Wang CL, Zeng QY. Molecular evolution of trehalose-6-phosphate synthase (TPS) gene family in Populus, Arabidopsis and rice. PLoS One. 2012:7(8):e42438 10.1371/journal.pone.0042438 PMC3414516 22905132

[kiad066-B117] Zacharaki V , Ponnu J, Crepin N, Langenecker T, Hagmann J, Skorzinski N, Musialak-Lange M, Wahl V, Rolland F, Schmid M. Impaired KIN10 function restores developmental defects in the *Arabidopsis trehalose 6-phosphate synthase1* (*tps1*) mutant. New Phytol. 2022:235(1):220–233 35306666 10.1111/nph.18104 PMC9320823

[kiad066-B118] Zhai Z , Keereetaweep J, Liu H, Feil R, Lunn JE, Shanklin J. Trehalose 6-phosphate positively regulates fatty acid synthesis by stabilizing WRINKLED1. Plant Cell. 2018:30(10):2616–2627 30249634 10.1105/tpc.18.00521 PMC6241258

[kiad066-B119] Zhai Z , Liu H, Shanklin J. Phosphorylation of WRINKLED1 by KIN10 results in its proteasomal degradation, providing a link between energy homeostasis and lipid biosynthesis. Plant Cell. 2017:29(4):871–889 28314829 10.1105/tpc.17.00019 PMC5435435

[kiad066-B121] Zhang Y , Primavesi LF, Jhurreea D, Andralojc PJ, Mitchell RA, Powers SJ, Schluepmann H, Delatte T, Wingler A, Paul MJ. Inhibition of SNF1-related protein kinase1 activity and regulation of metabolic pathways by trehalose-6-phosphate. Plant Physiol. 2009:149(4):1860–1871 19193861 10.1104/pp.108.133934 PMC2663748

[kiad066-B120] Zhang ZP , Deng Y, Song X, Miao M. Trehalose-6-phosphate and SNF1-related protein kinase 1 are involved in the first-fruit inhibition of cucumber. J Plant Physiol. 2015:177:110–120 25723473 10.1016/j.jplph.2014.09.009

