# Supplementary material for: In vivo protein kinase activity of SnRK1 fluctuates in Arabidopsis rosettes during light-dark cycles
Source: Plant Physiol. 2023 Feb 2;192(1):387–408. doi: 10.1093/plphys/kiad066 (PMC10152665; doi:10.1093/plphys/kiad066)
Supplement: kiad066_Supplementary_Data [file kiad066_supplementary_data.zip › Supplemental Files.pdf]

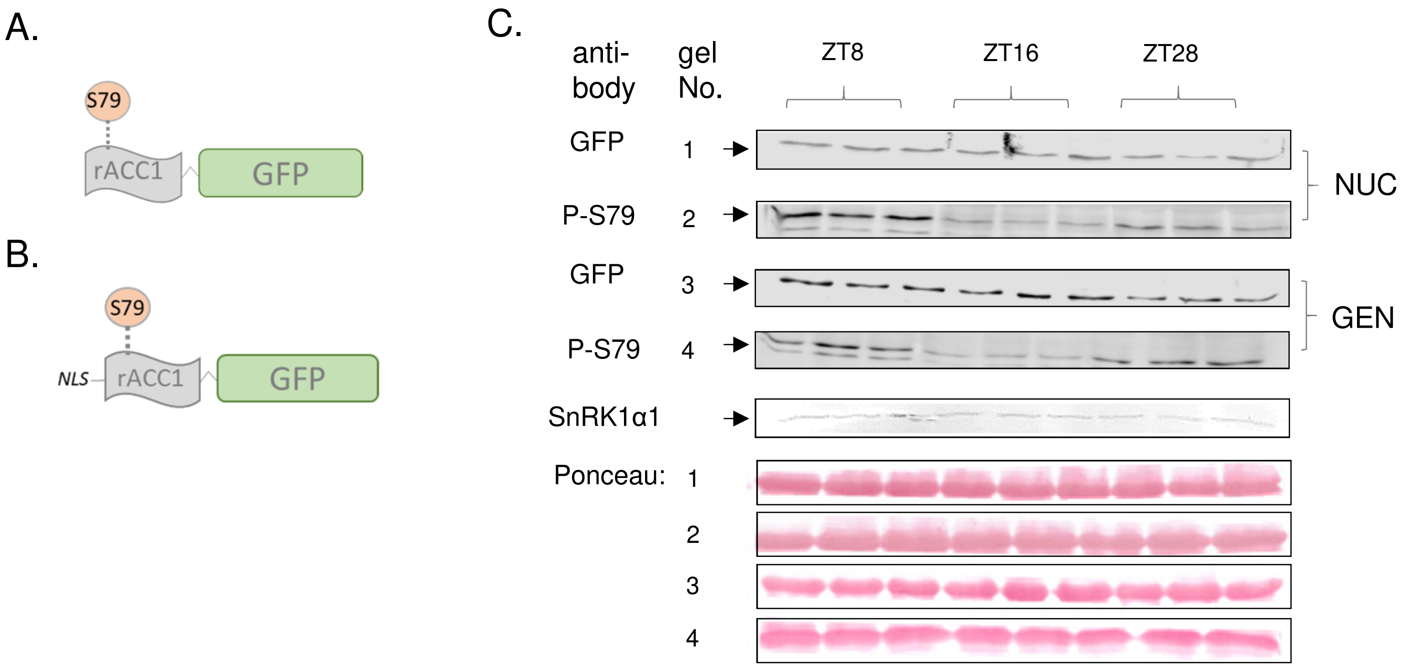

Supplemental Figure S1: *In-vivo* SnRK1 activity assay. The homozygous transgenic SnRK1 reporter lines were generated by Sanagi et al. (2021). The principle is based on sequence recognition homology between the three homologous kinases: mammalian AMPK, plant SnRK1 and yeast SNF1, as described in Deroover et al. (2016). In brief, a 57-aa peptide surrounding the Ser79 phosphorylation site of rat acetyl-CoA carboxylase 1 (rACC1) was fused to GFP, cloned into the pCB302 expression vector and transformed into Arabidopsis thaliana (Col-0). Two constructs were used: (A) a general reporter (GEN) and (B) a nuclear (NUC) reporter that includes the SV40 nuclear localization signal (see Figure S2 for localization data). (C) Quantification of SnRK1 activity: plants were grown in equinoctial growth conditions (12-h light / 12-h darkness) with 160 µmol m-2 s-1 irradiance (as described in Methods). Whole rosettes were harvested at the indicated times and immediately quenched in liquid nitrogen. Three biological replicates were harvested at each time point (four or five plants from the same pot were pooled for each biological replicate). About 12 µg of soluble proteins from each replicate were electrophoresed on an SDS-polyacrylamide gel (12% w/v), electro-blotted onto a nitrocellulose membrane. Replicate membranes were probed with anti-phospho-acetyl CoA carboxylase (pACC; S79), anti-GFP or anti-SnRK1α1 antibodies. Protein bands were quantified using the Image Studio Live 5.2 software (LI-CORE). The signal ratio pACC/GFP was determined to normalize for differences in loading and construct expression and used as readout of SnRK1 activity. A 4-h extended night (ZT28) sample was loaded in every gel to normalize for technical variance. Loaded samples: 1-3: ZT8, 4-6: ZT16 (night), 7-9: ZT28 (extended night). Protein loading was visualized by staining with Ponceau Red (the gel number indicates which set of Ponceau Red stained images corresponds to which SnRK1 reporter construct and antibody).

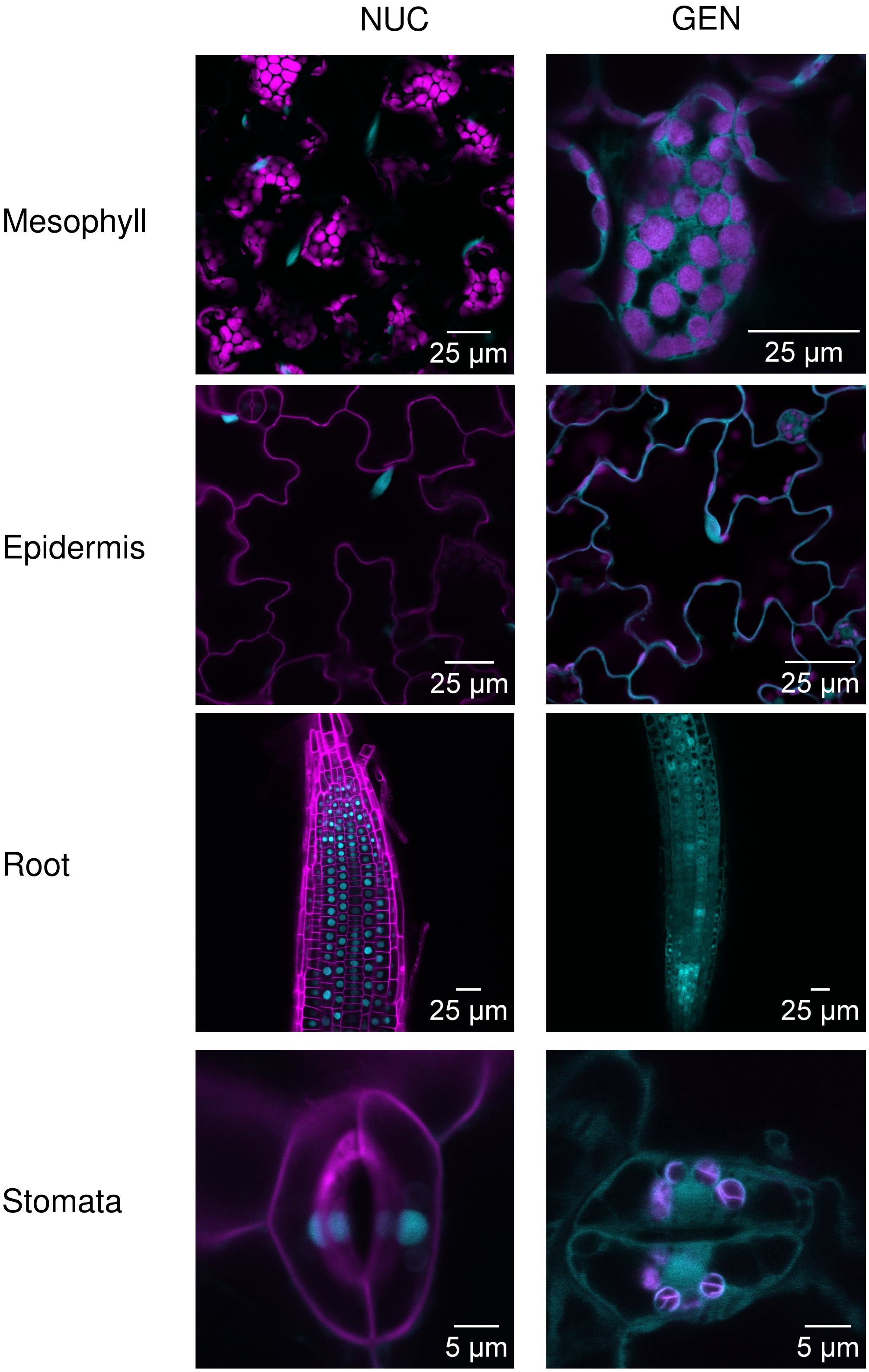

Supplementa1 Figure S2: Localization of reporter peptides by confocal microscopy.
Seedlings of NUC and GEN lines were grown on MS plates for 6 days and GFP signal was visualized by confocal microscopy, as described in Methods. NUC seedlings were further treated with propidium iodide (PI) to stain the cell wall. GEN samples were not stained with PI, to avoid overlapping signals between PI and plastids (chlorophyll). The figures show merged images of GFP and chlorophyll autofluorescence from the indicated tissues.

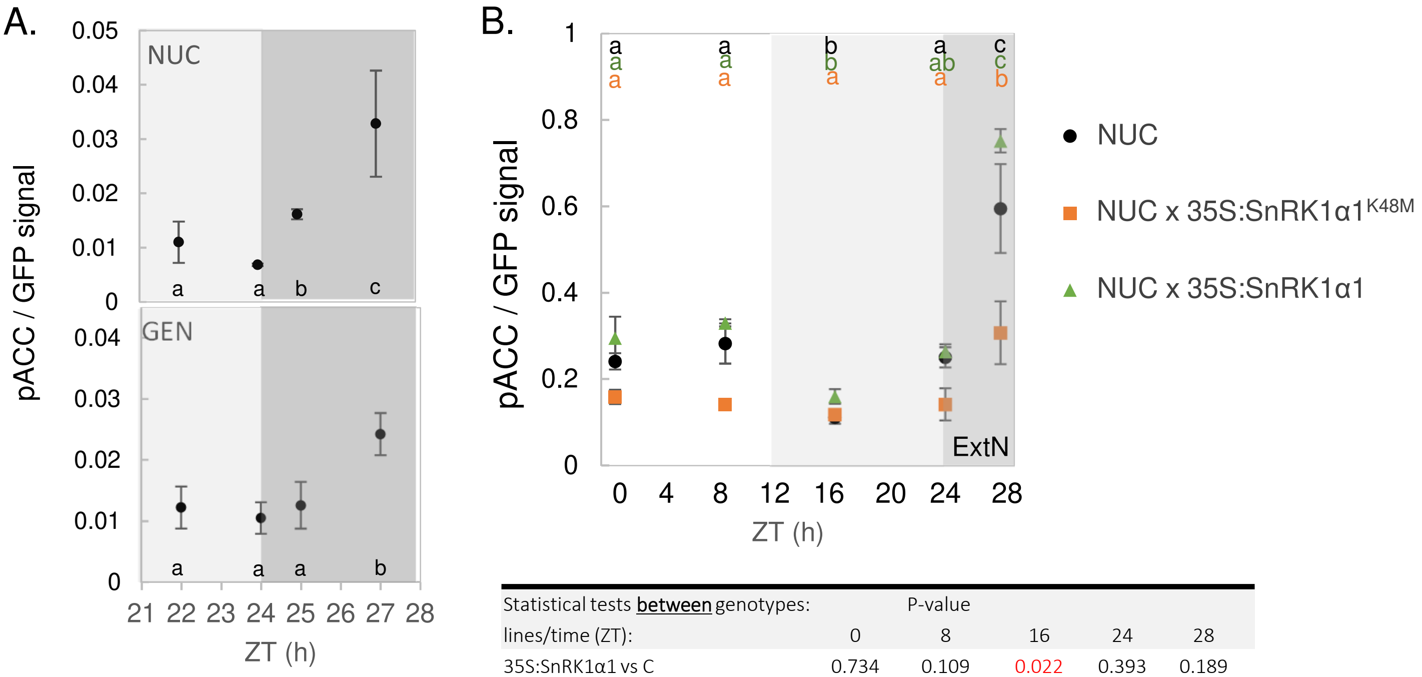

Statistical tests <u>between</u> genotypes:	P-value				
lines/time (ZT):	0	8	16	24	28
35S:SnRK1α1 vs C	0.734	0.109	0.022	0.393	0.189
35S:SnRK1α1^{K48M} vs C	0.023	0.001	0.892	0.003	0.006
35S:SnRK1α1 vs 35S:SnRK1α1^{K48M}	0.002	0.001	0.004	0.001	<0.001

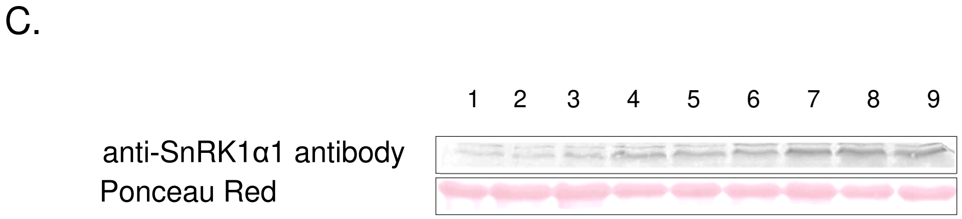

Supplemental Figure S3: Validation of the NUC and GEN constructs in whole Arabidopsis rosettes
(A) Extended darkness. NUC and GEN lines were grown in long day (16-h light / 8-h dark) conditions, with 160 µmol m^{-2} s^{-1} irradiance, for 22 days after sowing (22 DAS). On the day of harvest, plants were subjected to 3 h of extended night to activate SnRK1, and sampled at: ZT 20, 22, 24, 25, and 27. Immunoblots were performed as described in Methods and in Supplementary Figure S1C. Top -NUC, bottom – GEN. Letters represent statistical analysis (one-way ANOVA, followed by pairwise multiple comparison post-testing using the Holm-Sidak method, P<0.05). Results are shown as mean ± SD (n=2-4 biological replicates). See also Supplementary Dataset S1 – Exp.1. (B-C) High and low activity SnRK1 mutants. (B) NUC (control C), NUC x 35S:SnRK1^{K48M} and NUC x 35S:SnRK1α1 (i.e., overexpressing and dominant negative SnRK1 mutant lines crossed to NUC) were grown in equinoctial growth conditions (12-h light / 12-h dark) with 160 µmol m^{-2} s^{-1} irradiance for 22 DAS. Plants were harvested throughout day 23 after sowing at 8-h intervals and after 4 h of extended night (ExtN). Four replicates (each of 3-5 plants) were harvested at each time point. Immunoblots were performed as described in Methods and in Supplementary Figure S1C. Results are shown as mean ± SD (n=4 biological replicates). Statistical analysis: significance was tested separately for each line using one-way ANOVA, followed by pairwise multiple comparison post-testing using the Holm-Sidak method (P<0.05). Letters represent significant changes between different times for a given genotype. The statistical analysis for comparison between lines at a given time point is presented in the table, with significant (P<0.05) differences highlighted in red. See also Supplementary Dataset S1 – Exp.2. (C) Immunoblot assay using anti-SnRK1α1 antibody to confirm expression of the introduced SnRK1α1 and SnRK1^{K48M} proteins in the overexpression lines. All samples were harvested from the respective lines at ZT28 (4-h extended night): 1-3, NUC; 4-6, NUC x 35S:SnRK1α1^{K48M}; 7-9 NUC x 35S:SnRK1α1.

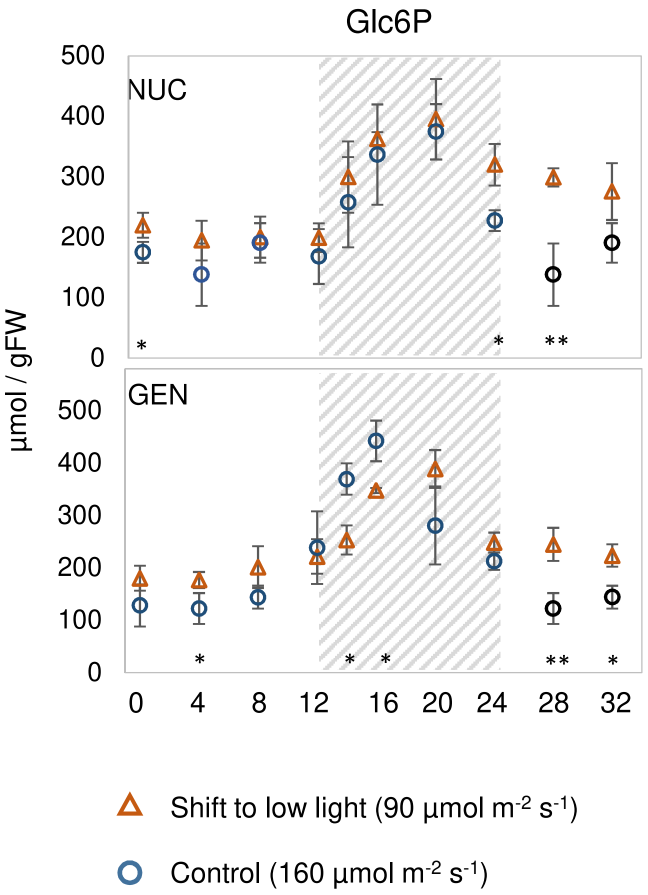

Supplemental Figure S4: Glc6P levels after a shift at dawn to continuous low light (supplemental to Figure 3).

Rosette Glc6P levels in NUC (upper panel) and GEN (lower panel) following transfer at ZT0 to continuous low light (90 µmol m^{-2} s^{-1}) compared to a control grown at the same time in standard conditions (12-h photoperiod, 160 µmol m^{-2} s^{-1} irradiance). The dashed grey background represents the night for control samples and the subjective night for treated samples Results are shown as mean ± SD (n=3-4 biological replicates). Control data points at ZT28 and ZT32 were not measured and therefore replicated from ZT4 and ZT8, respectively. Statistical analysis: one-way ANOVA, followed by pairwise multiple comparison post-testing using the Holm-Sidak method. Asterisks represent significant differences ($P<0.05$) between lines at a given time point. Data are from the same experiment shown in Figure 3, and are provided in Supplemental Dataset S1, experiment 4.

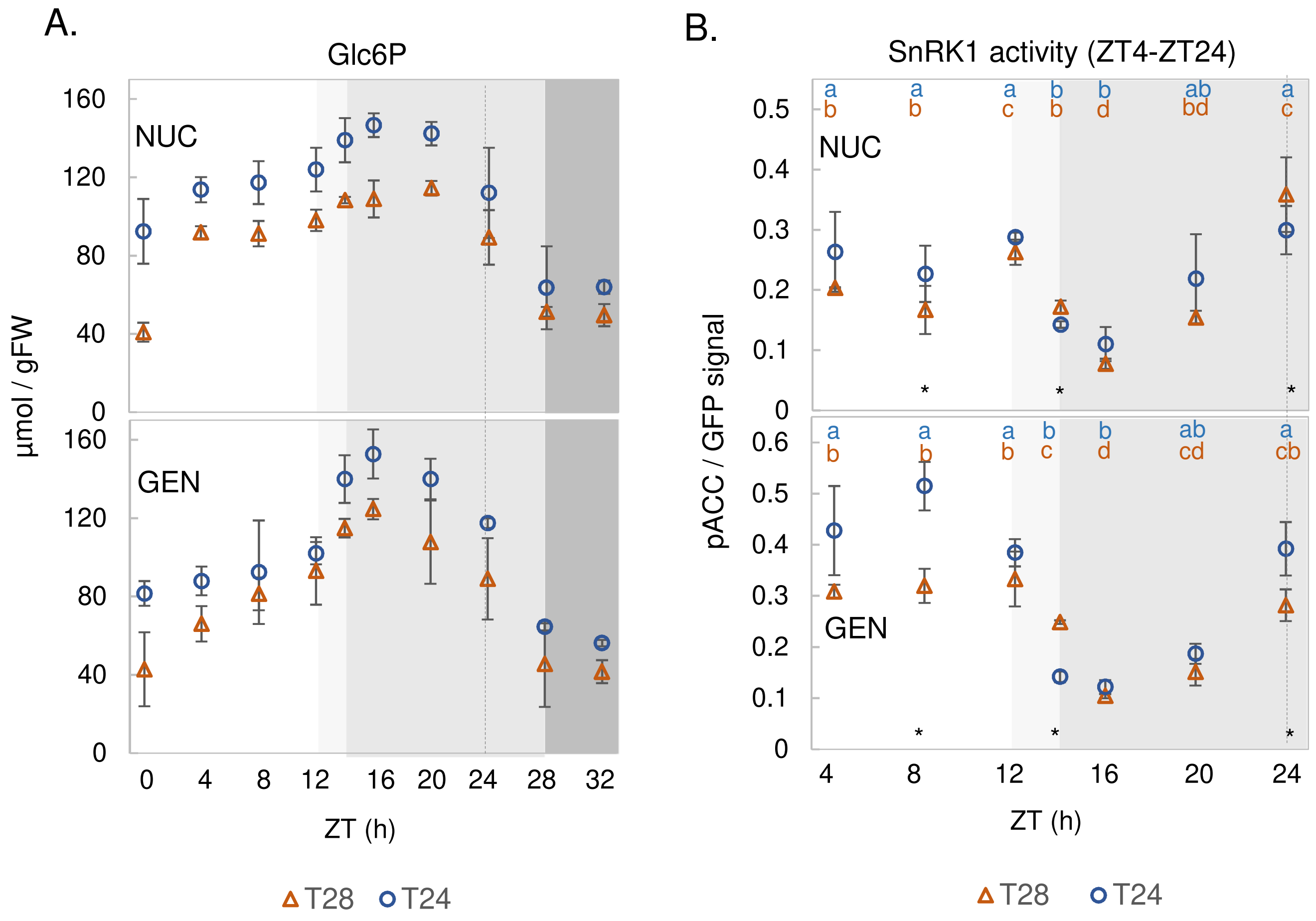

Supplemental Figure S5: T28 and T24 cycles; diel Glc6P levels and comparison of SnRK1 activity after excluding time points affected by C starvation (supplemental to Figure 4).

(A) Rosette Glc6P levels (measured enzymatically) from the T28 cycle experiment (described in Figure 4; Supplementary Dataset S1 Exp.5) for the NUC (upper) and GEN (lower) reporter lines. The T24 data from ZT0 to TZ24 is identical to the control treatment in the continuous low light treatment of Figure 3 (control B), with time points added for plants that were left in the dark from ZT24 onwards and harvested at ZT28 and ZT32. The vertical dotted line indicates the time at which control plants were transferred to continued darkness. **(B) Comparison of SnRK1 activity between T28 and the T24 control, excluding ZT0, ZT28 and ZT32 when plants were C-starved in the T28 cycle.** Upper panel NUC line, lower panel, GEN line. In (A) and (B), results are shown as mean ± SD (n=3 biological replicates), and the white background represents times when plants were in the light in both T24 control and T28 cycles, pale grey denotes darkness in the T24 control and light in the T28 cycle, mid-grey denotes darkness in both T-cycles (, and dark grey represents extended night in both T cycles. Statistical analysis was done by three separate one-way ANOVA tests, followed by pairwise multiple comparison post-testing using the Holm-Sidak method (P< 0.05): (i) comparing changes with time within the control (T24) line, indicated by blue letters; (ii) comparing changes with time within the T28 line, indicated by orange letters; and (iii) comparing between T24 and T28 samples at a given time point, indicated by asterisks.

A.

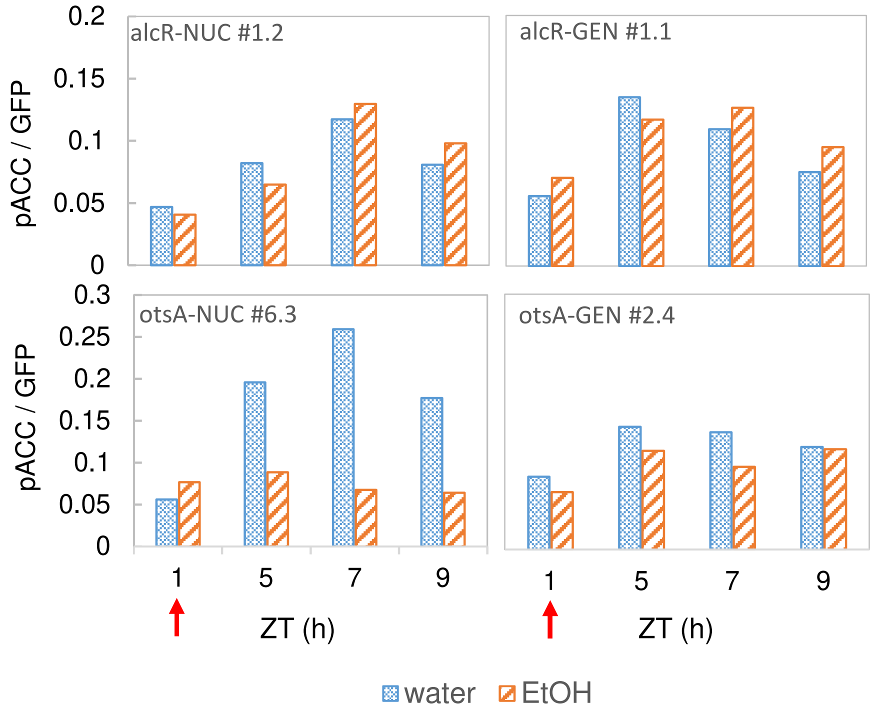

B.

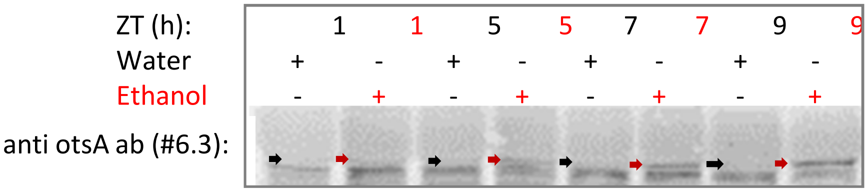

Supplemental Figure S6: Response of NUC and GEN phosphorylation in crosses with a line containing inducible bacterial TPS (supplemental to Figure 5).

NUC and GEN reporter lines were crossed with an ethanol-inducible otsA (bacterial TPS) line and with the empty vector (alcR) control line, as described in Methods. Selected homozygous plants (#6.3 NUC x iotsA; #2.4 GEN x iotsA, alcR #1.1-1.2) were grown in long day conditions (16-h light / 8 h dark) under 160 µmol m-2 s-1 illumination for 20 days. On day 21 after sowing, plants were sprayed at ZT1 (red arrow) with either water (mock induction control) or with 2% (v/v) ethanol (EtOH) to induce expression of the otsA protein and transiently increase Tre6P levels. Two plants (whole rosettes) were harvested and pooled as representative samples at 4, 6 and 8-h after water/EtOH spraying. (A) changes in SnRK1 phosphorylation activity upon otsA (TPS) induction. The ratios between the ethanol-induced and water control were determined for each time point, and then combined across all time points to test for significance in a given line. The differences were significant (Student's t-test, $P<0.05$) for #6.3 NUC x iotsA but not for #2.4 GEN x iots or the control alcR lines. (B) Immunoblot to confirm the inducible accumulation of otsA after spraying with ethanol in line #6.3 NUC x iotsA (indicated by the red arrows). The lower immunoreactive protein band seen in both induced and non-induced plants is also seen in leaf extracts from wild-type plants, and is due to non-specific binding of the antibody to a ~50-kDa protein that is probably the large subunit of Rubisco.

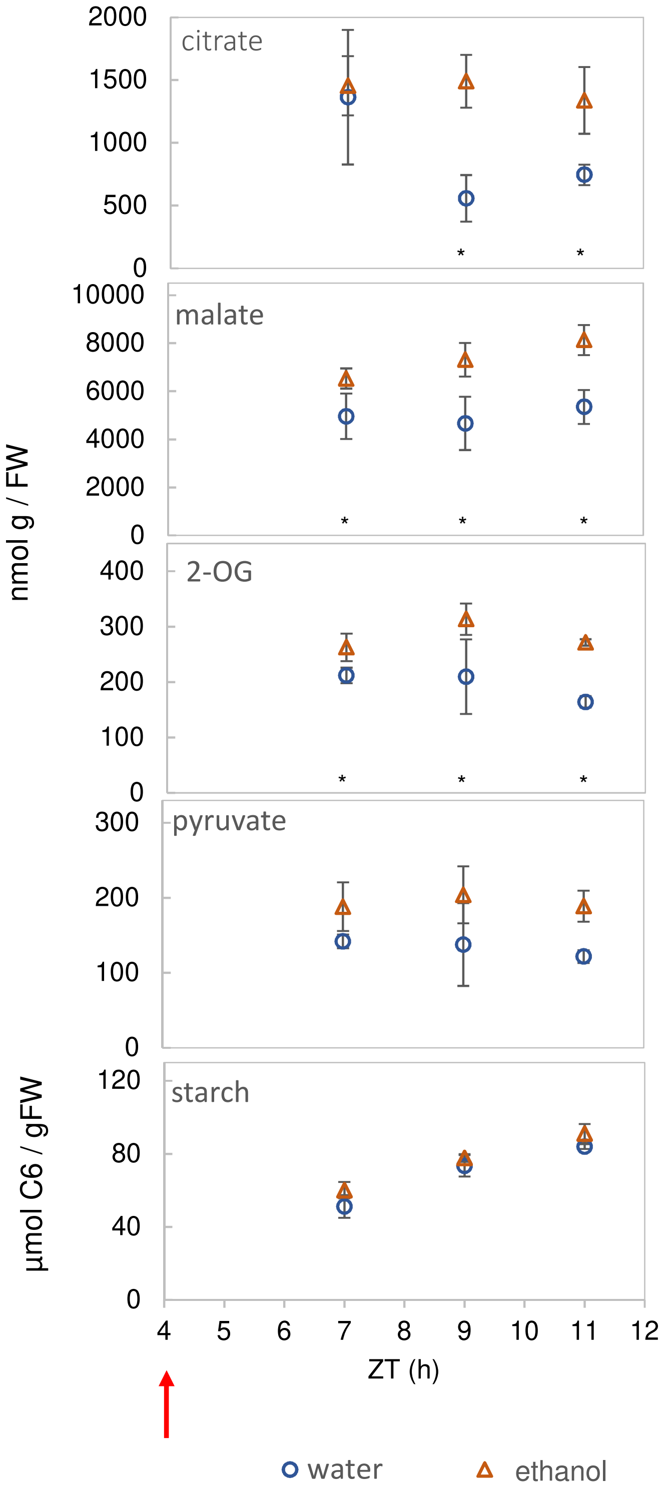

Supplemental Figure S7: Impact of transiently elevated Tre6P in the light period on the levels of selected metabolites (supplemental to Figure 5).

Line #6.3 (NUC x iotsA) was grown in equinoctial growth conditions (12-h light / 12 h dark) with 160 µmol m^{-2} s^{-1} irradiance for 20 days. On day 21 after sowing, plants were sprayed with either water or 2% (v/v) ethanol to induce the expression of bacterial TPS (otsA) at ZT4 (red arrow), and then harvested at ZT7, ZT9 and ZT11. Depicted are changes in selected metabolites. 2-OG, 2-oxoglutarate. Results are shown as mean ± SD (n = 3-4 biological replicates). Asterisks indicate statistically significant (P<0.05) differences between the water and ethanol-treated samples by one-way ANOVA followed by pairwise multiple comparison post-testing using the Holm-Sidak method. Data for additional metabolites from the same experiment are shown in Supplemental Dataset S1, experiment 7.

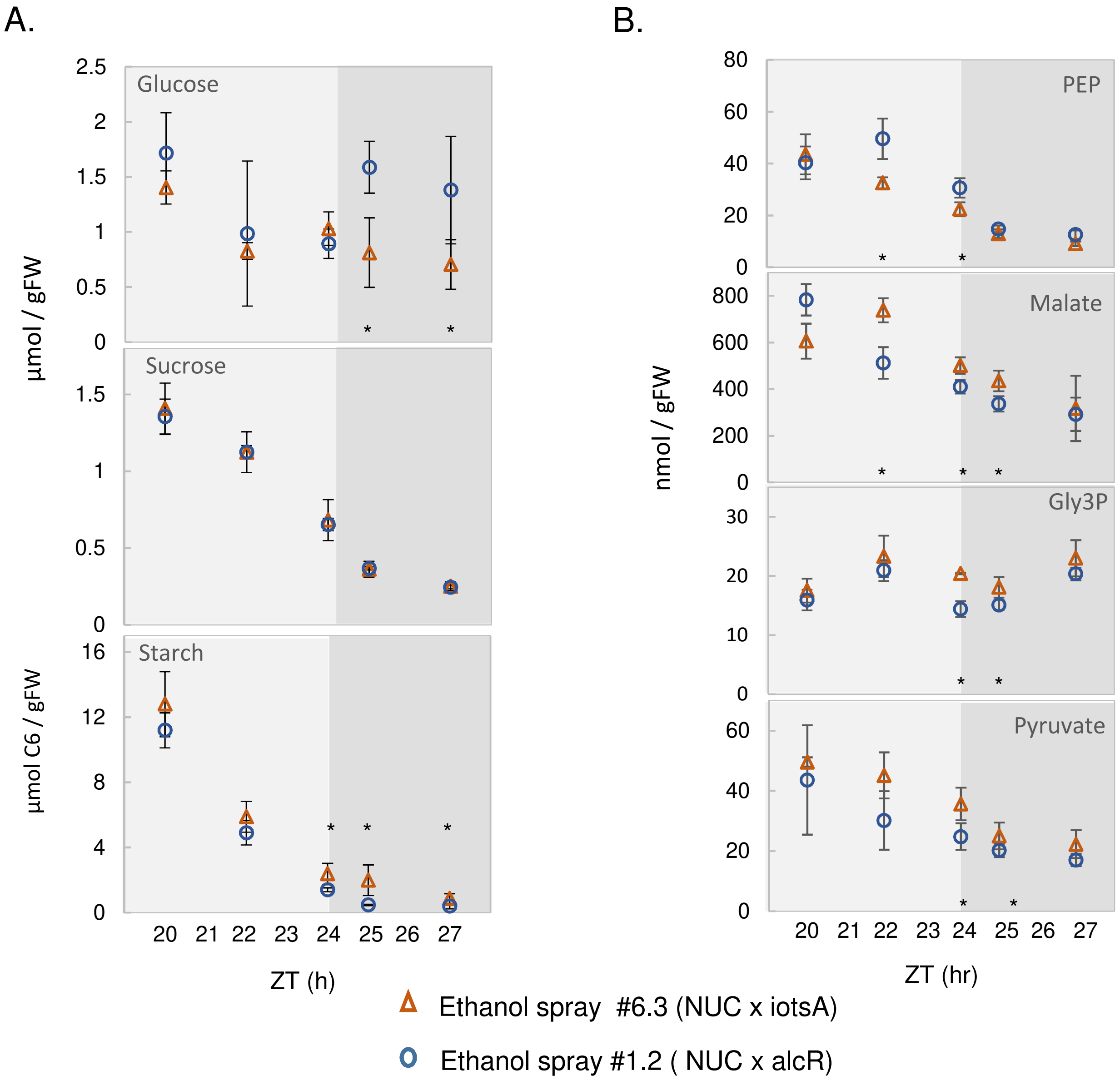

Supplemental Figure S8: Transient elevation of Tre6P at the end of the night slows down starch mobilization and leads to low glucose and high organic acid levels (supplemental to Figure 6).
Line #6.3 (NUC x iotsA) and control line #1.2 (NUC x alcR) were grown in short day conditions (6-h light / 18-h dark) with 160 µmol m^{-2} s^{-1} irradiance for 24 days. On day 25 after sowing, plants were sprayed at ZT20 with 2% (v/v) ethanol to induce expression of the otsA (TPS) protein, and thereby transiently increase Tre6P levels. After samples were harvested towards EN at ZT22 and ZT24, the remaining plants were transferred to extended darkness and harvested at ZT25 and ZT27 (as described in Figure 6). Four plants (whole rosettes) were pooled for each biological replicate. Rosettes were extracted for measurements of: (A) soluble sugars and starch, and (B) PEP, malate, glycerol 3-phosphate (Gly3P) and pyruvate. Data for additional metabolites are shown in Supplemental Figure S8, and Supplemental Dataset S1, experiment 7. Results are shown as mean ± SD (n = 3-4 biological replicates). Asterisks indicate significant ($P<0.05$) differences between the #6.3 (NUC x iotsA) and control lines according to one-way ANOVA with post-testing using the Holm-Sidak method.

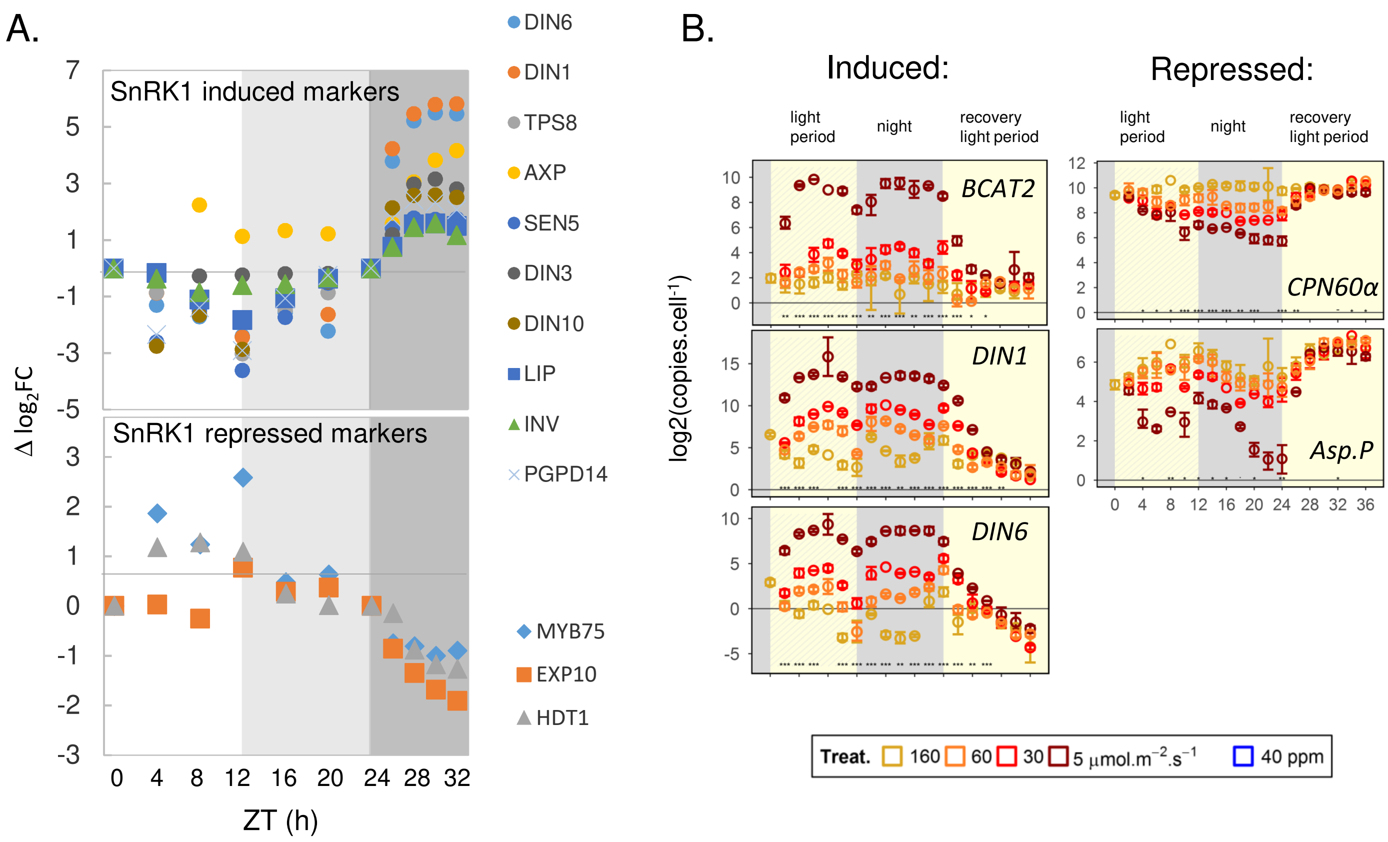

Supplemental Figure S9: Transcript abundance for SnRK1 marker genes during a diel cycle, after an extension of the night and after a sudden decrease in light intensity for one day.

Publicly available gene expression datasets were mined for selected genes that are commonly referred to as SnRK1 marker genes, based on their response to transient over-expression of SnRK1α1 in Arabidopsis mesophyll protoplasts (Baena-González *et al*., 2007). (A). Transcript abundance in wild-type Columbia-0 rosettes during a diel cycle in equinoctial conditions at an irradiance of 160 µmol m^{-2} s^{-1} and after an extension of the night. Expression data were extracted from the ATH1 microarray datasets reported in Usadel et al. (2008) and were normalized to the respective expression level at ZT0. (B) Transcript abundance in response to sudden low light day. Grey – night, dark grey – extended night. The experiment was conducted essentially as in Figure 3, except that a wider range of low light levels was used. Wild-type Columbia-0 plants were grown in equinoctial growth conditions (12-h photoperiod) with 160 µmol m^{-2} s^{-1} irradiance for 19 days. At the onset of the light period on day 20 after sowing, irradiance was reduced to 60, 30 or 5 µmol m^{-2} s^{-1} for the 12-h light period (low-light day). The plants were darkened at ZT12, and re-illuminated at ZT24 at the original growth irradiance of 160 µmol m^{-2} s^{-1} (recovery day). Results are shown as mean ± 95% confidence limits (n = 3 biological replicates, each containing five pooled rosettes). Asterisks indicate statistical significance of the combined response according to ANOVA: *** P=0, ** P<0.001, * P<0.01, P<0.05. P-values were adjusted using Benjamini & Hochberg false discovery rate across all time points in a given trait; the apparently significant difference for the *CPN60α* data at ZT32 (indicated by a dash) was rejected by the Tukey's honest significant difference post-test. Data were extracted from Supplemental Figure S6 in Moraes *et al*. (2019).

Supplemental Table S1: Correlations between metabolite levels and NUC or GEN phosphorylation in unperturbed diel cycles, performed separately for the light period and the night (supplemental to Table 1 and Figure 1).

R^2 and *P* values are shown for linear regression analyses performed separately on data from the three independent NUC control experiments and the two independent GEN control experiments plotted in Figure 1. Control A, B and C refer to the equinoctial diel control in Figure 2, Figure 3, and Supplemental Figure S3B, respectively (for data, see Supplemental Dataset S1, experiment 3, experiments 4-5 and experiment 2, respectively). Regressions were performed using data for individual replicates from the light period (ZT4, ZT8, ZT12) or the night (ZT16, ZT20, ZT24). Linear regression analysis was performed using the least squares method. The column +/- indicates whether the slope of the regression is positive (+) or negative (-). Significant correlations are highlighted in red ($P<0.05$). Individual regression plots are provided in Supplemental Dataset S2.

Experiment	Reporter	Metabolite	Day			Night		
			R^2	*P*-value	+/-	R^2	*P*-value	+/-
**Control A**	NUC	Tre6P	0.010	0.232	+	0.108	0.232	-
	NUC	Glc6P	0.035	0.558	-	0.595	2.7×10^{-4}	-
	NUC	Glc1P	0.126	0.257	-	0.609	6.0×10^{-4}	-
	NUC	Starch	0.000	1.000	-	0.822	3.2×10^{-6}	-
	NUC	Sucrose	0.000	0.973	+	0.760	2.3×10^{-5}	-
	NUC	Glucose	0.004	0.854	+	0.280	0.043	-
	GEN	Tre6P	0.240	0.181	-	0.347	0.044	-
	GEN	Glc6P	0.184	0.249	-	0.478	0.013	-
	GEN	Glc1P	0.099	0.410	-	0.243	0.103	-
	GEN	Starch	0.139	0.322	-	0.547	0.006	-
	GEN	Sucrose	0.241	0.180	-	0.348	0.004	-
	GEN	Glucose	0.172	0.267	+	0.275	0.080	+
**Control B**	NUC	Tre6P	0.152	0.300	+	0.441	0.007	-
	NUC	Glc6P	0.053	0.550	-	0.114	0.219	-
	NUC	Glc1P	0.251	0.169	-	0.364	0.017	-
	NUC	Starch	0.052	0.554	+	0.644	0.001	-
	NUC	Sucrose	0.124	0.352	+	0.514	0.006	-
	NUC	Glucose	0.082	0.454	+	0.006	0.808	-
	GEN	Tre6P	0.154	0.108	-	0.656	4.5×10^{-4}	-
	GEN	Glc6P	0.112	0.378	-	0.536	0.003	-
	GEN	Glc1P	0.122	0.356	-	0.707	1.7×10^{-4}	-
	GEN	Starch	0.003	0.892	-	0.740	7.9×10^{-5}	-
	GEN	Sucrose	0.249	0.171	-	0.824	7.4×10^{-6}	-
	GEN	Glucose	0.461	0.044	+	0.148	0.175	-
**Control C**	NUC	Tre6P	0.022	0.644	-	0.278	0.066	-
	NUC	Glc6P	0.022	0.811	+	0.251	0.057	-
	NUC	Glc1P	0.012	0.737	+	0.078	0.113	-
	NUC	Starch	0.001	0.969	+	0.686	7.3×10^{-5}	-
	NUC	Sucrose	0.056	0.458	+	0.011	0.702	-
	NUC	Glucose	0.001	0.917	-	0.131	0.168	-

Supplemental Table S2: **Correlations between metabolite levels (supplemental to Table 1 and Figure 1).**

R^2 and *P*-values are shown for linear regression analyses performed separately on data from the three independent control experiments with the NUC line and two independent experiments with the GEN line shown in Figure 1 (12-h photoperiod, 160 µmol m^{-2} s^{-1} irradiance). Regressions were performed using data for individual replicates from the complete diel cycle (ZT0 to ZT24). The linear regression analysis was done by using the least squares method. The column +/- indicates whether the slope of the regression is positive (+) or negative (-). Significant correlations are highlight in red (*P*<0.05), and metabolite pairs highlighted in bold were significantly correlated in all three NUC experiments and in the two independent GEN experiments (not presented). Individual plots are provided in Supplemental Dataset S2.

**NUC**		**Control A**			**Control B**			**Control C**		
		**R^2**	***P*-value**	**+/-**	**R^2**	***P*-value**	**+/-**	**R^2**	***P*-value**	**+/-**
Tre6P	Glc6P	0.532	$1.1x10^{-5}$	+	$1x10^{-4}$	0.885	-	0.287	0.006	+
Tre6P	Glc1P	0.340	0.001	+	0.007	0.697	+	0.147	0.048	+
**Tre6P**	**Sucrose**	0.400	$3.1x10^{-4}$	+	0.821	$2.7 x10^{-9}$	+	0.247	0.010	+
**Tre6P**	**Starch**	0.372	0.001	+	0.676	$1.5 x10^{-6}$	+	0.432	$2.7 x10^{-4}$	+
**Glc1P**	**Glc6P**	0.860	$1.3x10^{-12}$	+	0.654	$2.2 x10^{-6}$	+	0.279	0.007	+
Glc6P	Glucose	0.011	0.604	+	0.011	0.637	-	0.048	0.284	+
Glc6P	Sucrose	0.645	$2.7x10^{-7}$	+	$4x10^{-4}$	0.924	+	0.529	$3.8 x10^{-5}$	+
Glc6P	Starch	0.586	$2.1x10^{-6}$	+	0.001	0.885	+	0.695	$2.3 x10^{-7}$	+
Glc1P	Starch	0.601	$1.3 x10^{-6}$	+	0.051	0.300	+	0.089	0.138	+
**Sucrose**	**Starch**	0.642	$3.0 x10^{-7}$	+	0.775	$3.1 x10^{-8}$	+	0.474	$1.0 x10^{-4}$	+
Glucose	Starch	0.011	0.537	+	0.094	0.156	+	0.019	0.491	+

**GEN**		**Control A**			**Control B**		
		**R^2**	***P*-value**	**+/-**	**R^2**	***P*-value**	**+/-**
Tre6P	Glc6P	0.532	$1.1 x10^{-5}$	+	$1x10^{-4}$	0.885	-
Tre6P	Glc1P	0.340	0.001	+	0.007	0.697	+
Tre6P	Sucrose	0.400	$3.1 x10^{-4}$	+	0.821	$2.7 x10^{-9}$	+
Tre6P	Starch	0.372	0.001	+	0.676	$1.5 x10^{-6}$	+
Glc1P	Glc6P	0.860	$1.3 x10^{-12}$	+	0.654	$2.2 x10^{-6}$	+
Glc6P	Glucose	0.011	0.604	+	0.011	0.637	-
Glc6P	Sucrose	0.645	$2.7 x10^{-7}$	+	$4x10^{-4}$	0.924	+
Glc6P	Starch	0.586	$2.1 x10^{-6}$	+	0.001	0.885	+
Glc1P	Starch	0.601	$1.3 x10^{-6}$	+	0.051	0.300	+
Sucrose	Starch	0.642	$3.0 x10^{-7}$	+	0.775	$3.1 x10^{-8}$	+
Glucose	Starch	0.011	0.537	+	0.094	0.156	+

Supplemental Table S3: Correlations between changes in metabolite levels and changes in NUC or GEN phosphorylation (supplemental to Table 1 and Figure 1) in control treatments A, B and C.

R^2 and *P*-values are shown for linear regression analyses performed separately on data from the three independent NUC or the two independent GEN control experiments described in Figure 1 (see also legends of Table 1 and Supplemental Table S1). Changes in activity were calculated as the difference between absolute levels (averages) of two consecutive time points, divided by the time difference (per hour). The linear regression analysis was performed using the least squares method. Significant correlations are highlighted in red ($P<0.05$). The column +/- indicates whether the slope of the regression is positive (+) or negative (-). Individual regression plots are provided in Supplemental Dataset S2

Reporter	Metabolite	R^2	*P*-value	+/-
NUC	Tre6P	0.003	0.845	+
NUC	Glc6P	0.252	0.029	-
NUC	Glc1P	0.399	0.004	-
NUC	Starch	0.037	0.429	+
NUC	Sucrose	0.163	0.087	+
NUC	Glucose	0.009	0.706	-
GEN	Tre6P	0.295	0.068	-
GEN	Glc6P	0.617	0.002	-
GEN	Glc1P	0.369	0.028	-
GEN	Starch	0.010	0.743	-
GEN	Sucrose	0.121	0.244	-
GEN	Glucose	0.019	0.657	+

Supplemental Table S4: Correlations between metabolite levels and NUC or GEN phosphorylation in growth regimes that differed from the equinoctial T24 cycle (supplemental to Figures 3 and 4). Continuous (90) is the experiment reported in Figure 3 and Supplemental Dataset S1 experiment 4, in which plants were grown in a 12-h photoperiod at 160 µmol m^{-2} s^{-1} irradiance and then transferred at dawn to continuous irradiance at 90 µmol m^{-2} s^{-1} for 32 h. Regressions were performed using data from individual replicates across the entire time series from ZT0 to ZT32. T28 denotes the experiment reported in Figure 4 and Supplemental Dataset S2 experiment 5, in which plants were grown in a 14-h light / 14-h dark cycle. Regression analyses was performed using data from individual replicates on all times in light dark cycle (i.e. from ZT0 to ZT28). T28 (ZT4-ZT24) shows an additional analysis from the T28 experiment, using only time points between ZT4-ZT24 to eliminate possible interference due to C starvation at ZT0 and ZT28 (see Figure 4). This analysis was restricted to sugars, starch and Glc6P because no LC-MS/MS data were available for Tre6P and Glc1P. For data from both experiments, linear regression analysis was performed using the least squares method. The column +/- indicates whether the slope of the regression is positive (+) or negative (-). Significant correlations are highlighted in red ($P<0.05$). Individual plots are provided in Supplemental Dataset S2. n.d., metabolite not determined in this experiment. Individual regression plots are provided in Supplemental Dataset S2

Reporter	Metabolite	Continuous (90)			T28			T28 (ZT4-ZT24)		
		R^2	*P*-value	+/-	R^2	*P*-value	+/-	R^2	*P*-value	+/-
NUC	Tre6P	0.462	$2.6x10^{-4}$	-	n.d.	n.d.		n.d.	n.d.	
NUC	Glc6P	0.414	0.001	-	0.889	$1.9x10^{-13}$	-	0.366	0.004	-
NUC	Glc1P	0.228	0.018	-	n.d.	n.d.		n.d.	n.d.	
NUC	Starch	0.012	0.972	-	0.534	$1.5x10^{-5}$	-	0.255	0.020	-
NUC	Sucrose	0.057	0.243	-	0.687	$9.4x10^{-8}$	-	0.151	0.082	-
NUC	Glucose	0.059	0.738	-	0.027	0.416		0.002	0.857	-
GEN	Tre6P	0.337	0.004	-	n.d.	n.d.		n.d.	n.d.	
GEN	Glc6P	0.314	0.005	-	0.674	$2.8x10^{-7}$	-	0.496	0.001	-
GEN	Glc1P	0.262	0.013	-	n.d.	n.d.		n.d.	n.d.	
GEN	Starch	0.083	0.322	-	0.477	$6.8x10^{-5}$	-	0.019	0.550	-
GEN	Sucrose	0.021	0.511	-	0.693	$7.2x10^{-5}$	-	0.065	0.265	-
GEN	Glucose	0.097	0.099	-	0.260	0.007	-	0.115	0.132	+

Supplemental Table S5: **Summary of correlation analysis between changes in SnRK1 NUC or GEN activity and changes in metabolite levels (supplemental to Figures 3 and 4).**

Changes in activity were calculated as the difference between absolute levels (averages) of two consecutive time points, divided by the time difference (per hour) as described in Table S3. See Supplemental Table S4 for details of the experiments and the time spans used for the correlation analysis. For the T28 data set, the analysis was restricted to sugars, starch and Glc6P because no LC-MS/MS data were available for Tre6P and Glc1P. Linear regression analysis was performed using the least squares method. Significant correlations (*P*<0.05) are highlighted in red. The column +/- indicates whether the slope of the regression is positive (+) or negative (-). n.d., metabolite not determined in this experiment. Individual regression plots are provided in Supplemental Dataset S2

Reporter	Metabolite	Continuous (90)			T28			T28 (ZT4-ZT24)		
		R^2	*P*-value	+/-	R^2	*P*-value	+/-	R^2	*P*-value	+/-
NUC	Tre6P	0.542	0.059	-	n.d.	n.d.		n.d.	n.d.	
NUC	Glc6P	0.550	0.056	-	0.873	0.001	-	0.444	0.148	-
NUC	Glc1P	0.390	0.134	-	n.d.	n.d.		n.d.	n.d.	
NUC	Starch	0.114	0.459	+	0.191	0.279	-	0.051	0.668	-
NUC	Sucrose	0.044	0.650	+	0.320	0.144	-	0.005	0.899	+
NUC	Glucose	0.131	0.426	-	0.015	0.771	-	0.026	0.758	+
GEN	Tre6P	0.403	0.125	-	n.d.	n.d.		n.d.	n.d.	
GEN	Glc6P	0.460	0.094	-	0.537	0.039	-	0.485	0.124	-
GEN	Glc1P	0.422	0.114	-	n.d.	n.d.		n.d.	n.d.	
GEN	Starch	0.304	0.199	-	0.178	0.298	-	0.030	0.744	-
GEN	Sucrose	0.097	0.496	-	0.391	0.097	-	0.001	0.950	+
GEN	Glucose	0.204	0.309	-	0.334	0.134	-	0.074	0.602	+

Supplemental Table S6: **Correlation analysis between metabolite levels and NUC phosphorylation following transient elevation of Tre6P in the light period (supplemental to Figure 5 and Supplemental Dataset S2 experiment 7).**

R^2 and *P*-values are shown for regression analyses after treatment with ethanol, or in the water (mock induction) control. Linear regression analyses were performed using the least squares method on data for individual replicates. The column +/- indicates whether the slope of the regression is positive (+) or negative (-). Significant correlations ($P<0.05$) are highlighted in red. Individual regression plots are provided in Supplemental Dataset S2

**Reporter**	**Metabolite**	**Water**		**Ethanol**	
		R^2	*P*-value	R^2	*P*-value
NUC	Tre6P	0.671	0.004	0.012	0.763
NUC	Glc6P	0.243	0.148	0.140	0.287
NUC	Glc1P	0.153	0.263	0.047	0.547
NUC	Starch	0.004	0.869	0.091	0.398
NUC	Sucrose	0.028	0.819	0.105	0.361
NUC	Glucose	0.000	0.983	0.328	0.083

Supplemental Text S1. Correlations between *in-vivo* SnRK1 activity and metabolites in continuous light, in T28 cycles and in experiments where TPS is induced.

In-vivo SnRK1 activity correlates with Tre6P and especially Glc6P and Glc1P in a recurring equinoctial diel cycle (see main text, Table 1 and Supplemental Table S1). To test whether these correlations are found under perturbations of the diel cycle, we utilized the data from experiments where plants were shifted to continuous irradiance at 90 µmol m^{-2} s^{-1} µmol m^{-2} s^{-1} (Figure 3, Supplemental Dataset S1 Exp.4) or grown in a T28 cycle (Figure 4, Supplemental Dataset S1 Exp.5), and performed additional regressions between diel SnRK1 NUC and GEN phosphorylation and metabolite levels (Supplemental Table S3; see Supplemental Dataset S2 file for plots).

In the shift to continuous lower irradiance, Tre6P, Glc6P and Glc1P were significantly and negatively correlated to SnRK1 NUC (R^2 = of 0.46, 0.41, and 0.23, respectively) and GEN (R^2 of 0.34, 0.31, and 0.26, respectively). No significant correlation was observed with starch, sucrose or glucose. Additional regressions were performed using derivatives (average change over time). These showed no significant correlation of SnRK1 activity with any metabolite (Supplemental Table S4).

For plants growing in a T28 cycle (Fig. 4), the analysis was more restricted. Metabolites were only measured enzymatically and not by LC-MS/MS, so Tre6P and Glc1P were absent from these analyses. To avoid regressions that were driven by the very high phosphorylation of NUC and GEN at ZT0 and ZT28 (low C; Figure 4), the analyses were performed on two different sets of values; once over the full 28-h cycle and the second over a narrowed time scale from ZT4-ZT24. Regressions of absolute diel levels over the full 28-h cycle revealed significant correlation between NUC phosphorylation and Glc6P, starch and sucrose levels, and between GEN phosphorylation and Glc6P, starch, sucrose and glucose levels. The more focused analysis using ZT4-ZT24 showed significant correlation between NUC phosphorylation and Glc6P and starch levels, and between GEN phosphorylation and Glc6P levels. Similarly, regressions of average change over time intervals (derivatives) revealed a significant correlation between both NUC and GEN phosphorylation and Glc6P, using the full 28-h cycle (Supplemental Table S4).

Regression analysis was also performed for the experiments with lines containing iTPS. These calculations were limited by the relatively small number of time points. When Tre6P was increased by TPS induction in the light period, (Fig. 5), NUC phosphorylation correlated negatively with Tre6P in the control (R^2 = 0.67, p = 0.004) but not in the induced TPS line (R^2 = 0.12, p = 0.76) (Supplemental Table S6). This is consistent with the idea that SnRK1 activity is regulated by a network including Tre6P, and that this network responds to decrease the contribution of Tre6P when Tre6P levels are artificially elevated. Glc was unrelated to SnRK1 in the control and negatively related after ethanol spraying (R^2

= 0 and 0.33, respectively), but the latter negative correlation was still only very weakly significant (*P* = 0.08). When Tre6P was elevated by TPS induction towards the end of the night and harvesting was continued into an extended night, NUC phosphorylation correlated across successive time points negatively with Tre6P, Glc6P, Glc1P, sucrose and starch (not shown). This might reflect this experiment having been conducted at a time when C is becoming depleted and there is a progressive and strong decrease in the levels of many C metabolites that are potentially involved in regulation of SnRK1.

The ‘statistical power’ of correlation analyses performed on the three control time series (whole diel cycles) was larger than those performed on the perturbation, because three independent time series from different experiments were available for the standard diel cycle and because the perturbations often had fewer time points per time series. This means that the analyses in the control conditions can better distinguish between primary and secondary (i.e. indirect) correlations.
